# Using tea nanoclusters as β-lactamase inhibitors to cure multidrug-resistant bacterial pneumonia: A promising therapeutic strategy by Chinese materioherbology

**DOI:** 10.1016/j.fmre.2021.11.019

**Published:** 2021-11-25

**Authors:** Ziao Zhou, Jun Li, Lei Tan, Xiangmei Liu, Yufeng Zheng, Zhenduo Cui, Changyi Li, Kelvin Wai Kwok Yeung, Zhaoyang Li, Yanqin Liang, Shengli Zhu, Shuilin Wu

**Affiliations:** a Biomedical Materials Engineering Research Center, Collaborative Innovation Center for Advanced Organic Chemical Materials Co-constructed by the Province and Ministry, Hubei Key Laboratory of Polymer Materials, Ministry-of-Education Key Laboratory for the Green Preparation and Application of Functional Materials, School of Materials Science & Engineering, Hubei University, Wuhan 430062, China; b School of Materials Science & Engineering, Peking University, Beijing 100871, China; c School of Materials Science & Engineering, the Key Laboratory of Advanced Ceramics and Machining Technology by the Ministry of Education of China, Tianjin University, Tianjin 300072, China; d Stomatological Hospital, Tianjin Medical University, No. 12, Qixiangtai Road, Heping District, Tianjin 300070, China; e Department of Orthopaedics & Traumatology, Li Ka Shing Faculty of Medicine, The University of Hong Kong, Pokfulam, Hong Kong 999077, China

**Keywords:** Materioherbology, Antibacterial, β-Lactam antibiotic, Pneumonia, Tea nanoclusters

## Abstract

β-lactamase, a kind of hydrolase in multi-drug resistant pathogens, can hydrolyze β-lactam antibiotics and make these kinds of antibiotics invalid. Small-molecular inhibitors about the enzyme and their mechanism are widely investigated but they may result in unavoidable adverse reactions and drug-resistance. Herein, we propose a new therapeutic strategy of Chinese materioherbology, in which herbal medicine or traditional Chinese medicinal herbs can be employed as biological functional materials or refreshed/excited by means of materialogy. Natural tea nanoclusters (TNCs) were extracted from tea to inhibit β-lactamase. Different from the mechanism of small-molecular inhibitors inhibiting enzymes by binding to the corresponding active sites, the TNCs as a cap cover the protein pocket and create a spatial barrier between the active sites and antibiotics, which was named “capping-pocket” effect. TNCs were combined with amoxicillin sodium (Amo) to treat the methicillin-resistant *Staphylococcus aureus* (MRSA) pneumonia in mice. This combinatorial therapy remarkably outperforms antibiotic monotherapy in reducing MRSA infections and the associated inflammation in mice. The therapeutic strategy exhibited excellent biosafety, without any side effects, even in piglets. Hence, TNCs have great clinical value in potentiating β-lactam antibiotic activity for combatting multi-drug resistant pathogen infections and the "pocket capping" effect can guide the design of new enzyme inhibitors in near future.

## Highlights

• A new nanostructure of TNCs with much stronger enzyme inhibitory effect.
• A new mechanism to inhibit enzymes by a “pocket capping” effect.
• Zapping β-lactam antibiotics into action and eradicating MRSA induced pneumonia.

## Graphical abstract

For the first time, we propose the concept of pocket capping, in which tea nanoclusters with right size as a cap directly block protein pockets, preventing small molecules from entering active areas in protein pocket and interacting with the protein. Therefore, β-lactam antibiotics were not hydrolyzed by β-lactamase. Natural tea nanoclusters (TNCs) were prepared and combined with conventional β-lactam antibiotics to effectively cure MRSA-induced pneumonia in mice via inhalation.

**Figure:**
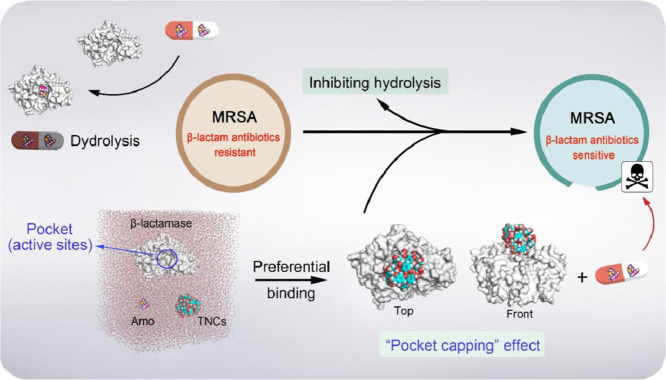

## 1 Introduction

In medical institutions and communities, *Staphylococcus aureus* is one of the most common pathogens that cause infectious diseases [1], [2], [3]. The infections may be severe and cause local purulent infections, such as purulent infections of skin and soft tissues; respiratory infections and pneumonia; digestive tract infections and systemic blood system infections, which become major threats to the public health [4]. For pneumonia, in particular, more than two million children under 5 years of age die from this disease each year in the world, which is far more than the number of deaths from measles, malaria, and acquired immune deficiency syndrome [5]. Although pneumonia affects the health of children worldwide, the problems are concentrated mainly in developing countries, such as those in Africa, Asia, and South America [6]. Conventional antibiotics show great potential in addressing this problem [7, 8]. However, in recent years, the emergence and rapid spread of multi-drug resistant bacteria including methicillin-resistant *Staphylococcus aureus* (MRSA) have led to the difficult treatment of bacteria-induced pneumonia because these bacteria are insensitive to almost all conventional antibiotics. Thus, conventional antibiotics have gradually become ineffective and will be eliminated [9], [10], [11], [12].

In order to treat MRSA pneumonia, one approach is to find new antibacterial drugs, but it takes 10 years or more for a new drug to be developed clinically at the expense of a great deal of cost [13]. Conversely, it only takes bacteria no more than two weeks to develop resistance to a new drug [14]. Another approach is to use vancomycin to treat severe infections or increase the concentration of traditional antibiotics, such as amoxicillin, penicillin, and ampicillin, which cause serious adverse reactions, inducing damages more than the pneumonia itself [15]. In view of these, it may save much time and financial cost if we can find a way to re-sensitize MRSA clinically to conventional antibiotics without any side effects.

To address this problem, diverse strategies have been developed; one of these is the use of smart drug delivery systems to target the site of infection and improve the efficiency of conventional antibiotics [16]. This treatment can increase the concentration of the infected site and reduce the toxic side effects of the infected site. Nanomaterials have been widely used to carry antibiotics for targeted treatment of infections, and nanomaterials themselves can also interact with bacterial membranes to improve the efficiency of antibiotic treatment [17]. However, there was an inevitable problem. Precise targeting of drugs in the human body was relatively difficult to achieve and drug delivery antibiotics have problems with targeting, such as drug off-target [18, 19]. Another was the use of antibacterial adjuvants [20], which can inhibit bacterial resistance to conventional antibiotics such as β-lactam antibiotics, the most commonly prescribed antibiotics in the basic shopping list recommended by the World Health Organization [21], and effectively increase the sensitivity of bacteria to drugs and restore their clinical utility. The underlying mechanism of MRSA resistance to β-lactam antibiotics is a large expression of β-lactamase, which can hydrolyze β-lactam antibiotics [22]. However, owing to the unavoidable differences in biological toxicity and pharmacokinetics of synthetic antibacterial adjuvants, the application of these adjuvants is limited [23, 24].

**Fig. 1:**
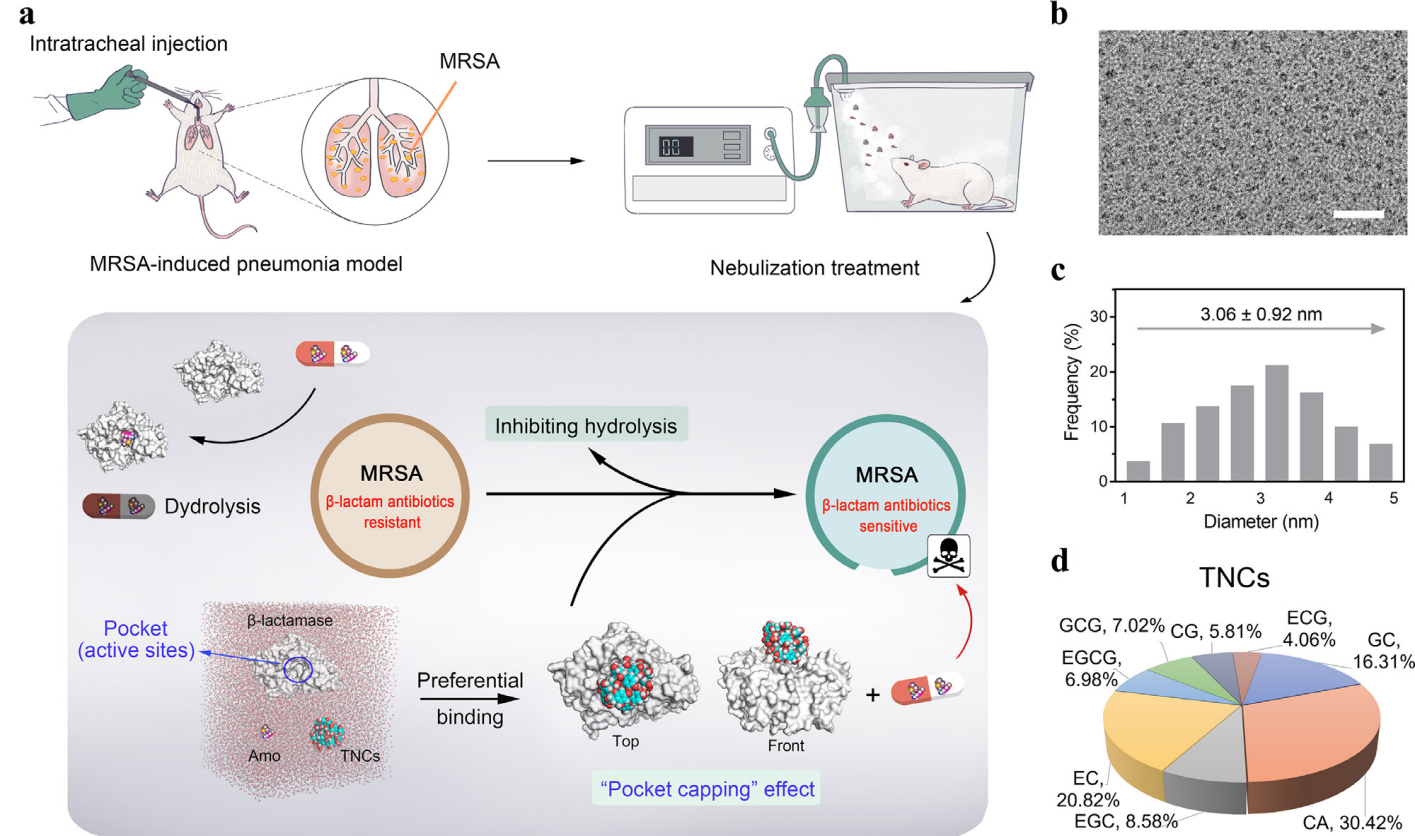
**Schema represents TNCs potentiating β-lactam antibiotics to treat mice with MRSA-induced pneumonia.** (a) Schematic of the MRSA-induced pneumonia model, the nebulization treatment using TNCs that potentiate β-lactam antibiotics, and the potentiating mechanism of β-lactam antibiotics by TNCs. (b) High-resolution transmission electron microscope (HR-TEM) image of the TNCs. Scale bar, 20 nm. (c) Size distribution of TNCs measured from the HR-TEM image. (d) Types and proportions of ingredients that make up TNCs using high-performance liquid chromatography coupled with triple-quadrupole tandem mass spectrometry (HPLC-MS-MS). Catechin (CA), Gallocatechin (GC), Catechin gallate (CG), Gallocatechin gallate (GCG), Epicatechin (EC), Epigallocatechin (EGC), Epicatechin gallate (ECG), Epigallocatechin gallate (EGCG).

Natural products have become a valuable source because of their sustainable features and good biosafety [25]. Tea is one of the most widely consumed beverages, and since ancient times, it has been regarded as a healthful beverage in traditional Chinese medicine [26]. Drinking tea has many health benefits, including anti-inflammatory, anti-oxidative, and antiviral effects, as well as the prevention of cardiovascular diseases and cancer [27, 28]. In this study, we propose a new therapeutic strategy of Chinese materioherbology, in which herbal medicine or traditional Chinese medicinal herbs can be employed as biological functional materials or refreshed/excited by means of materialogy. Specifically, a simple hydrothermal method was employed to prepare tea nanoclusters (TNCs), which was utilized to inhibit the activity of enzymes via a “pocket capping” effect. Once TNCs bound to the protein pocket of β-lactamase, TNCs served as capping (space barrier) between active residues and β-lactam antibiotics, hindering the binding of active residues and β-lactam antibiotics. Therefore, β-lactam antibiotics were not hydrolyzed by β-lactamase. β-lactam antibiotics successfully reached penicillin-binding proteins (PBPs) and inhibited the synthesis of bacterial cell walls, finally killing the bacteria [29]. The use of local inhalation to treat pneumonia in mice effectively avoided low drug utilization and side effects in other parts of the body. After the health of experimental mice was restored, no side effects were observed. Even in large animal experiments, such as piglets, TNCs did not show any side effects. In view of the low cost and excellent biosafety of TNCs and the wide use of β-lactam antibiotics clinically, we demonstrated that this strategy of TNCs potentiating β-lactam antibiotics has great potential for the worldwide treatment of MRSA-infected pneumonia with low medical cost while maintaining the best therapeutic effects (Fig. 1a).

## 2 Results

### 2.1 Characterization of TNCs and potentiation of β-lactam antibiotics to MRSA with the addition of TNCs

From the high-resolution transmission electron microscope image of the TNCs (Fig. 1b), we observed that the average diameter was about 3 nm (Fig. 1c). Next, high-performance liquid chromatography coupled with triple-quadrupole tandem mass spectrometry was utilized to analyze the compositions of the TNCs (Figs. 1d and S1–5). We compared the results of two independent experiments (A and B). There were only minor changes between group A and the group B (Fig. S1). The maximum change value (A-B) /A did not exceed 2%. These demonstrated that the reproducibility of the types and proportions of ingredients that make up TNCs was good.

**Fig. 2:**
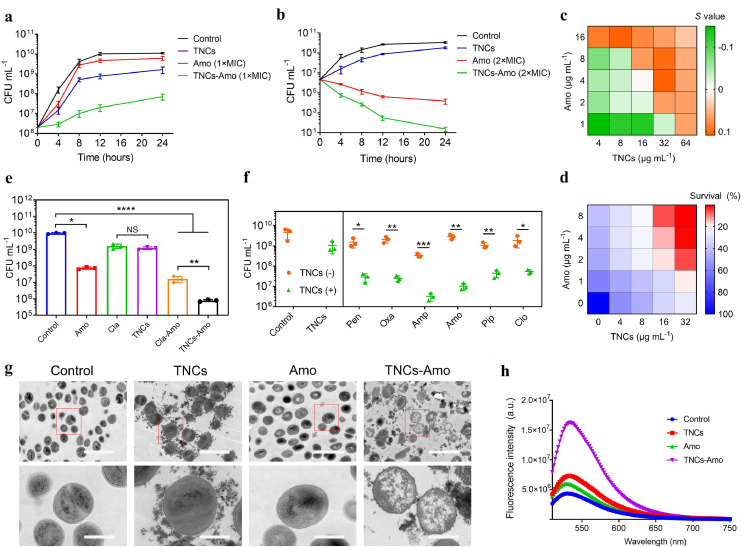
**TNCs potentiate β-lactam antibiotic activity *in vitro.*** (a) Growth curves of bacteria under treatment with TNCs, Amo (1 × MIC), and TNCs–Amo (1 × MIC) for 24 h. The initial concentration of the bacterial suspension is 2 × 10^6^ CFU mL^−1^. (b) Growth curves of bacteria under treatment with TNCs, Amo (2 × MIC), and TNC–Amo (2 × MIC) for 24 h. The initial concentration of the bacterial suspension is 2 × 10^6^ CFU mL^−1^. (c) The Bliss Independence model is used to evaluate synergistic interaction between TNCs and Amo. *S* > *0* indicates a synergistic interaction and *S* < *0* represents an antagonistic interaction. (d) Survival rate of bacteria treated with Amo at various concentrations combined with TNCs of different concentrations. (e) Comparison of TNCs’ ability to re-sensitize antibiotics with Cla’ s ability to re-sensitize antibiotics. (f) The antibacterial efficacy of Penicillin G potassium salt (Pen), Oxacillin sodium salt (Oxa), Ampicillin sodium salt (Amp), Amoxicillin sodium (Amo), Piperacillin sodium (Pip), and Cloxacillin sodium salt monohydrate (Clo) combined with TNCs after 8 h. (g) Comparison of bacterial transmission electron microscope (TEM) images in the presence of TNCs, Amo and TNCs–Amo. Scale bar (top) = 2 μm and scale bar (bottom) = 500 nm. (h) Fluorescence intensity of SYTOX Green of MRSA treated with TNCs, Amo, and TNCs–Amo. Test conditions: *λ*_ex_ = 485 nm, *λ*_em_ = 525 nm. In (a) (b) (e) and (f), the experiments were repeated three times independently with alike results, *P < 0.05, **P < 0.01, ***P < 0.001, ****P < 0.0001.

The main multidrug resistance (MDR) mechanism in MRSA to β-lactam antibiotics is over-expression of β-lactamase to hydrolyze β-lactam antibiotics [30]. We selected one of the most commonly used β-lactam antibiotics in clinics, and we combined amoxicillin sodium (Amo) at a concentration of 1 × MIC with TNCs (64 μg mL^−1^) to co-culture MRSA for 24 h. From the bacterial growth curves at different conditions (Fig. 2a), we can observe no obvious differences between the Amo (1 × MIC) and the control, which indicated that the Amo at this concentration was inefficient to MRSA. Amo slightly inhibited the growth of bacteria. However, after the combination of the TNCs with Amo, the bacterial growth curve became significantly slower than that of the other groups, and the CFU mL^−1^ at 24 h was reduced by about three orders of magnitude compared with the Amo (1 × MIC) group; this indicated that with the help of TNCs, ineffective Amo re-activated its vitality against MRSA. The concentration of the TNCs in Figs. 2a, and S5a, b was 64 μg mL^−1^ and the MIC of the TNCs was 64 μg mL^−1^. Amo, Oxa, Pen and TNCs at the concentration of 1 × MIC, TNCs had a better bacterial effect than Amo, Oxa and Pen. But if the concentration of Amo was increased from 1 × MIC to 2 × MIC and the concentration of TNCs was 1 × MIC (Fig. 2b), Amo had a better bacterial effect than TNCs. The bacterial growth curves of Amo and TNC–Amo (2 × MIC) decreased significantly compared with that of the control, and the CFU mL^−1^ of the combinatorial group at 24 h was reduced by three orders of magnitude compared with the free antibiotic group. Importantly, almost all bacteria were killed in the TNC–Amo (2 × MIC) group over 24 h. In addition, the bacterial growth curves of other β-lactam antibiotics of both Penicillin G potassium salt (Pen) and Oxacillin sodium salt (Oxa) combined with TNCs were investigated (Fig. S7a, b). The results demonstrated that MRSA had increased sensitivity to β-lactam antibiotics in the presence of TNCs.

In order to evaluate the combinatorial nature of TNCs with β-lactam antibiotics, the Bliss Independence model was employed [31, 32]. The enhancement effect of different concentrations of TNCs on different concentrations of antibiotics was investigated when combined to combat bacteria (Figs. 2c, and S7c, d). When the concentration of TNCs was the same, the increasing doses of antibiotics gave rise to an increase in potentiation degree. We could also draw the same conclusion by comparing the CFU mL^−1^ of the Amo group with that of the combinatorial group at different concentrations of Amo after coculturing them with MRSA for 24 h (Fig. S8). The results further highlighted that TNCs can potentiate β-lactam antibiotics. In Fig. 2c, only when TNCs was 64 μg mL^−1^, the S values were all greater than 0, which proved that when TNCs was 64 μg mL^−1^, antibiotics had a synergistic effect with TNCs at different concentrations. Too high concentration of TNCs could increase the toxicity of tissues and organ. So, we selected the TNCs concentration of 64 μg mL^−1^ for the combination therapy. Next, we investigated whether the synergistic effect caused the MIC of the antibiotics to decline. From the testing criteria of MIC [33], we knew that bacterial survival rates below 20% was the MIC of antibiotics after combination with MRSA (5 × 10^5^ CFU mL^−1^) for 24 h. We used checkerboard-style assays to evaluate the MIC of antibiotics with a series of β-lactam antibiotics (Amo, Oxa, and Pen) in the presence of TNCs (Figs. 2d, and S9a, b). Each antibiotic with five different concentrations (0.5, 0.25, 0.125, 0.0625, and 0 × MIC) was combined with TNCs (32, 16, 8, 4, and 0 μg mL^−1^) to co-incubate with MRSA for 24 h. Our results showed that the MIC of antibiotics in the presence of TNCs (32 μg mL^−1^) was reduced by 16-fold compared with their original MIC. These data showed the significant potentiation of β-lactam antibiotics’ viability.

Clavulanate potassium (Cla) has been the first choice to inhibit β-lactamase in MRSA, and it has been clinically utilized with Amo to treat MRSA [34]. Therefore, in our work, we compared the therapeutic effect of TNCs and Cla combined with Amo at the same concentration against MRSA (Fig. 2e). The result showed that the CFU mL^−1^ of the TNCs–Amo group was reduced by 20-fold compared with that of the Cla–Amo (P < 0.01, n = 3) group. Therefore, in terms of re-sensitizing Amo, TNCs had far better performance than Cla at the same concentration. Given that TNCs can potentiate Amo, we explored whether TNCs can potentiate other β-lactam antibiotics, including Pen (1 × MIC), Amp (1 × MIC), Pip (1 × MIC), Clo (1 × MIC), and Oxa (1 × MIC). As we expected, the antibacterial performance of these five kinds of β-lactam antibiotics was all markedly improved compared with that of the free antibiotic groups in the presence of TNCs (Fig. 2f).

Next, transmission electron microscope was used to observe the morphologies of MRSA co-cultured with TNCs, Amo, and TNCs–Amo (Fig. 2g). TNCs were all adsorbed on the surface of the bacterial cell membrane. The bacteria in the Amo group still exhibited a smooth shape with little change. Almost all the structures inside the bacteria were destroyed in the combinatorial group, but the ones inside the bacteria of the other groups were intact. The images detected by a field emission scanning electron microscope also demonstrated the result except for the state inside the bacteria (Fig. S10). In addition, SYTOX Green membrane permeability assay exhibited that TNCs can permeate the cell membrane of MRSA (Figs. 2h and S11) [35]. Confocal laser scanning microscope images demonstrated that the TNCs-Amo group had stronger fluorescence than other groups and the permeability of the cell membrane was changed. MRSA treated with the antibiotic exhibited a smooth shape with little change due to the β-lactamase in the MRSA hydrolyzing the antibiotic. The antibiotic could not work, and the cell membrane was smooth. However, in the experiment of SYTOX green membrane permeability, because the TNCs inhibited the activity of β-lactamase in the TNCs-Amo group, the antibiotic could not be hydrolyzed. Antibiotics worked to cause the death of bacteria and changes in the permeability of cell membranes. So, the change in cell membrane permeability was due to the action of antibiotics.

### 2.2 β-Lactamase inactivation by TNCs *in vitro*

**Fig. 3:**
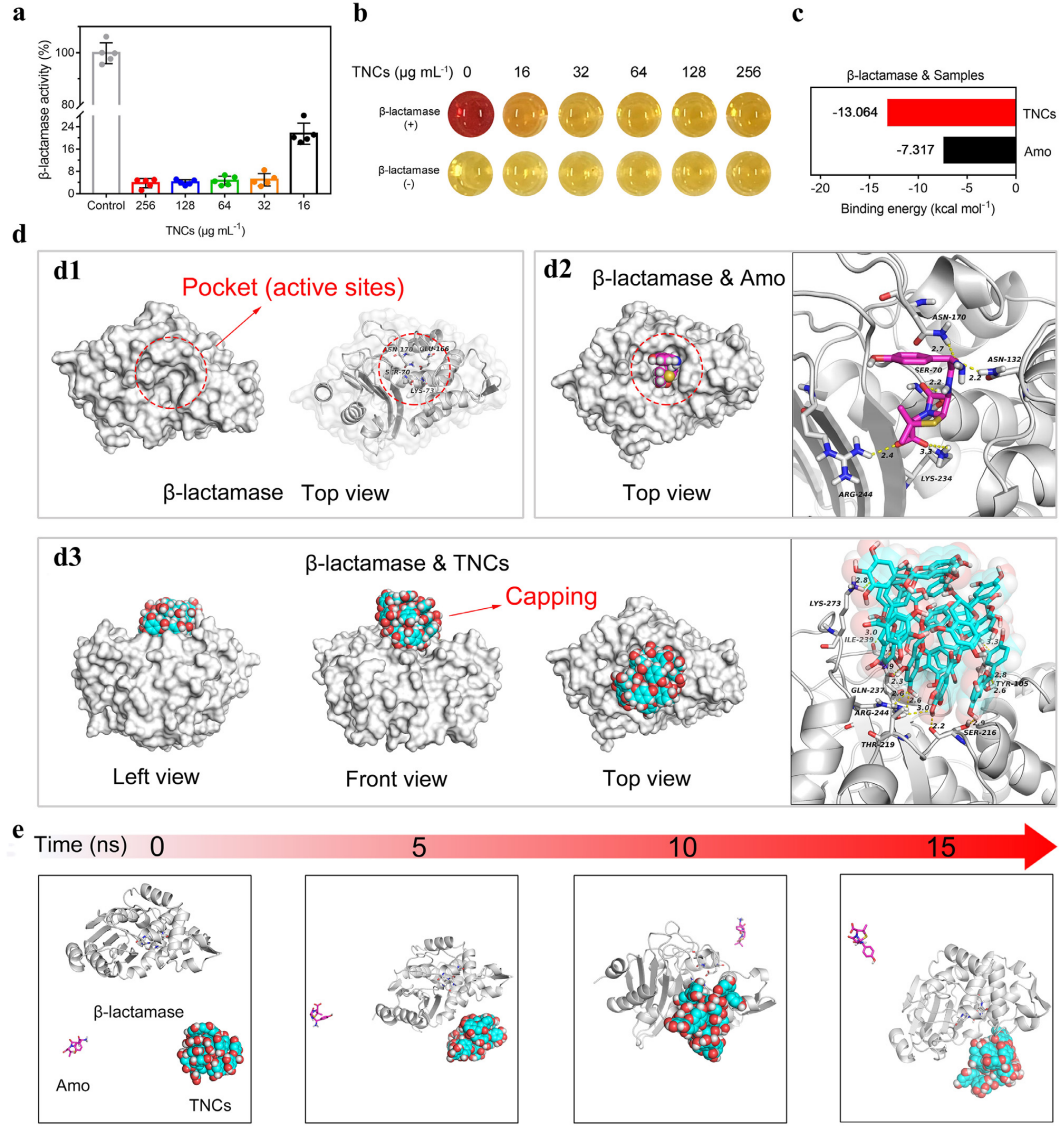
**Study of the mechanism of β-lactamase inactivation.** (a) Activity of β-lactamase treated with TNCs at various concentrations for 8 h. n = 5 independent experiments with similar results. (b) Optical pictures of residual β-lactamase tested by nitrocefin after the TNCs interacted with β-lactamase. The redder the color, the greater the β-lactamase. The original color of nitrocefin is yellow. (c) Binding capacity of β-lactamase to TNCs and Amo. (d) Pocket capping effect: (d1) The top view of β-lactamase and the location of pocket (active sites); (d2) The top view of β-lactamase & Amo and the active sites between Amo and β-lactamase; (d3) The three-view drawing of β-lactamase & TNCs and binding of TNCs to β-lactamase residues. The yellow dotted line represents the hydrogen bond. (e) Conformation changes of TNCs-Amo-β-lactamase at different time nodes (0, 5, 10 and 15 ns) during all atom molecular dynamics simulation.

To further investigate whether β-lactamase activity was inhibited by TNCs, TNCs were combined with β-lactamase and co-cultured for 8 h (Fig. 3a). Nitrocefin was used to evaluate the activity of β-lactamase [36]. We clearly observed that when the concentration of the TNCs was just only 16 μg mL^−1^, the β-lactamase activity was about 21%. When the concentration of the TNCs was increased to 32 μg mL^−1^ or more, the β-lactamase activity decreased below 10%. The corresponding color of the above mixtures showed that the color of the solution changed from red to yellow, indicating that the TNCs inhibited β-lactamase activity (Fig. 3b). The binding constant (K) of TNCs with β-lactamase measured by Isothermal Titration Calorimetry (ITC) was 1.12 × 10^5^ ± 6.73 × 10^4^ M^−1^. This result demonstrated that there was weak interaction between TNCs and β-lactamase (Fig. S12).

### 2.3 Mechanism of β-lactamase inactivation *via* simulations

To further explore the interaction of Amo and TNCs with β-lactamase at the molecular level, molecular docking was used. The binding free energy between TNCs and β-lactamase residues was lower than that of the Amo group, suggesting that the binding stability of TNCs with β-lactamase was much better than that of Amo (Fig. 3c). Next, the lowest energy binding modes of Amo and TNCs with β-lactamase were selected for visual analysis (Fig. 3d). The active sites of β-lactamase were located in the protein pocket (Fig. 3d1) [37]. From the top view of β-lactamase & Amo, Amo was embedded in the protein pocket and the carboxyl group in Amo formed hydrogen bond interactions with the key residues ARG244 and LYS234 in β-lactamase, resulting in the subsequent failure of Amo against MRSA (Fig. 3d2). From the lowest energy binding mode of β-lactamase & TNCs, TNCs completely blocked the pocket as capping due to the matching size of TNCs and the pocket, abundant contact surface, and hydrogen-bonding interaction. TNCs had strong hydrogen-bonding interactions with key residues TYR105, SER216, THR219, ARG244, GLN237, ILE239, and LYS273 around the protein pocket (Fig. 3d3). The key residues formed a dense hydrogen bond network with the abundant phenolic hydroxyl groups on the TNCs surface, which held the TNCs like a claw, thus making the β-lactamase bind to the TNCs stably and tightly. Consequently, TNCs can be used as capping to cover the protein pocket and invalidated the active residues. The capping prevented Amo from entering the binding sites, resulting in the inhibition of Amo hydrolysis by β-lactamase when the TNC–Amo system was used to kill MRSA.

To further explore whether TNCs can preferentially bind to β-lactamase in comparison with Amo, all atomic Molecular dynamics simulation (MDS) were performed for the competitive binding of TNCs and Amo with β-lactamase. The TNC–Amo–β-lactamase complex system was established with a positive triangular structure (Fig. S13). After the completion of the simulation (Supplementary Video 1), the conformation extraction and comparative analysis of the all atomic MDS demonstrated that during the MDS (Fig. 3e), the TNC gradually approached β-lactamase and bound to the enzyme after 5 ns. However, to study the binding stability of TNC and β-lactamase, the time of MDS was extended to 15 ns. As shown in Fig. S14, because the TNCs had better spatial matching (suitable size) and stronger interaction (hydrogen bond) with β-lactamase compared with Amo, the TNC–β-lactamase complex remained stable without dissociation. The TNC–β-lactamase complex exhibited a gradual reduction in binding energy and a more stable binding (Fig. S15). On the contrary, Amo did not bind to β-lactamase during the MDS, so calculation of binding energy could not be performed. These results demonstrated that compared with Amo, TNCs preferentially bound to β-lactamase when they competed for binding with β-lactamase and then covered the pocket in β-lactamase as a capping, resulting in β-lactam antibiotics unable to enter the pocket (active sites) to be hydrolyzed.

### 2.4 Toxicological assay and biosafety research in piglets

**Fig. 4:**
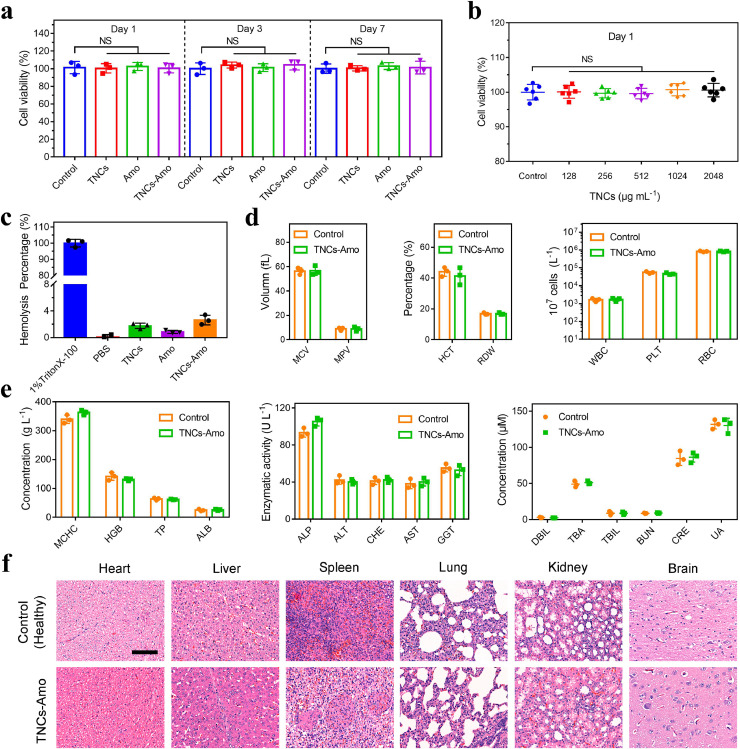
**Biosafety assay.** (a) The cell viability of A549 cells with TNCs, Amo, and TNCs–Amo was tested by LDH after 1, 3, and 7 days. (b) Cell viability of A549 cells treated with TNCs at various concentrations after co-culturing for 1 day (n = 5 independent experiments). (c) Hemolysis test of samples. (d, e) Biosafety study of TNCs–Amo in piglets. Hematology (mean corpuscular volume [MCV], mean platelet volume [MPV], hematocrit [HCT], red cell distribution width [RDW], white blood cell [WBC], platelets [PLT], red blood cell [RBC], mean corpuscular hemoglobin concentration [MCHC], and hemoglobin [HGB]), liver function (total protein [TP], albumin [ALB], alkaline phosphatase [ALP], alanine aminotransferase [ALT], cholinesterase [CHE], aspartic transaminase [AST], γ-glutamyl transpeptidase [GGT], and direct bilirubin [DBIL]), and kidney function (urea nitrogen [BUN], creatinine [CRE], and uric acid [UA]) were tested in piglets after nebulization with TNCs–Amo. (f) Hematoxylin and eosin (H&E)-stained images of tissue sections from piglets. Scale bar, 100 μm. In (a), (c), (d) and (e), the experiments were repeated three times independently with similar results.

Human alveolar basal epithelial (A549) cells were used to assess the toxicity of materials, and the viability of A549 cells was evaluated with lactate dehydrogenase assay [38, 39]. The control (no treatment), TNCs, Amo, and TNCs–Amo were co-incubated with A549 cells separately (Fig. 4a). After incubation for 1, 3, and 7 days, compared with the control, all groups exhibited high cell viability of over 95%. In addition, we also investigated the toxicity of different concentrations of TNCs at 1, 3, and 7 days (Figs. 4b and S16a, b). Even if the concentration of the TNCs was up to 2,048 μg mL^−1^, the cell viability was still over 95%, which indicated that the TNCs had good biosafety. All aforementioned groups, including the negative control (PBS) and the positive control (1% TritonX-100), were treated with red blood cells (Figs. 4c and S17). The result of the hemolytic assay showed that the hemolysis percentage of all groups, except the positive control, was below 4%. To test the biosafety and efficiency of TNCs–Amo against MRSA, we co-cultured MRSA and A549 cells together (Fig. S18a, b). These results proved that the combination of TNCs and Amo increased the cell viability even with MRSA infection.

Piglets and humans have great similarities in anatomy, genetics, and physiology [40]. To further investigate the biosafety of TNCs–Amo in vivo, the piglet model was utilized. Seven days after nebulization, hematology (mean corpuscular volume, mean platelet volume, hematocrit, red cell distribution width, white blood cell, platelets, red blood cell, mean corpuscular hemoglobin concentration, and hemoglobin) and liver functions (total protein, albumin, alkaline phosphatase, alanine aminotransferase, cholinesterase, aspartic transaminase, γ-glutamyl transpeptidase, and direct bilirubin) were used to evaluate the biosafety of TNCs–Amo in vivo (left and middle in Fig. 4d, e,). However, the experimental outcome was that an inappreciable gap could be observed in healthy piglets and piglets treated with TNCs–Amo. Renal functions (urea nitrogen, creatinine, and uric acid) were also tested, and no significant differences could be found between the healthy (no treatment) and the TNCs–Amo groups (Fig. 4e, right). Finally, no signs of abnormalities or lesions of the heart, spleen, lung, liver, kidney, and brain in hematoxylin and eosin (H&E) staining images at 7 days post-surgery were observed (Fig. 4f). These results provided evidence that the therapy had excellent biosafety and might be used for the therapy of large animals and even humans with MRSA-induced pneumonia.

### 2.5 Antibacterial studies and lung infection treatment *in vivo*

**Fig. 5:**
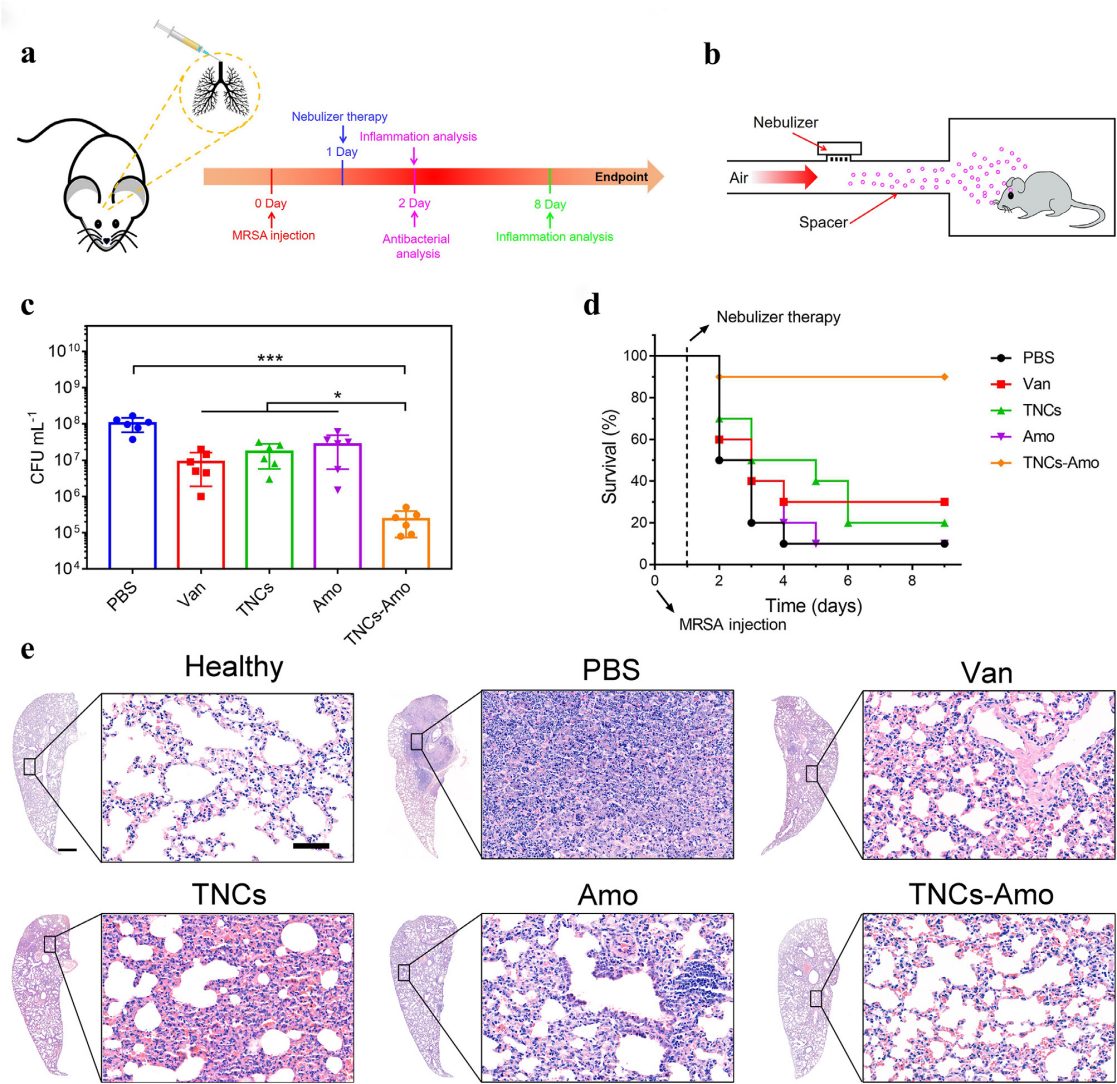
**TNCs enhance antibiotic therapy *in vivo.*** (a) Schematic of the infection and treatment protocol. (b) Pneumonitis treatment diagram for mice. (c) CFU of MRSA in infected lungs after treatment with PBS (control), Van, TNCs, Amo and TNCs–Amo. (d) Survival rate (n = 10) of mice inoculated intratracheally with 5 × 10^8^ CFU of MRSA with the following therapeutics: PBS, Van, TNCs, Amo, and TNCs–Amo. (e) H&E staining images of lungs in mice on day 8. Scale bar (left), 500 μm. Scale bar (right), 50 μm. In c, n = 5 biologically independent experiments. *P < 0.05, ***P < 0.001.

To further study the efficiency of this therapy and whether TNCs could increase the sensitivity of MRSA to β-lactam antibiotics *in vivo*, we used nebulization to treat a mouse model of acute pneumonia. A schematic protocol of this therapy was presented in Fig. 5a, b. In our animal model, MRSA (5 × 10^8^ CFU) was injected intratracheally into BALB/c mice to cause acute pneumonia [41]. To eradicate the MRSA from the lungs and restore the infected mice to health, we stably nebulized the mice with PBS, Amo, TNCs, and TNCs–Amo for 15 min twice a day. After nebulization, the lungs of all groups were assayed for residual MRSA (Fig. 5c). The TNC treatment alone reduced the CFU of MRSA by six-fold compared with the PBS group. Meanwhile, for the Amo group, the bacterial counts were just reduced by threefold compared with the PBS group. This was because of the drug resistance of the bacteria to Amo. Compared with the Amo group, the TNCs–Amo group showed a significant reduction in MRSA counts by two orders of magnitude (*P* < 0.05, n = 5), which proved that Amo activity was increased sharply with the addition of TNCs in vivo. Furthermore, with combinatorial therapy (TNCs–Amo), the viable MRSA in the lung dramatically decreased 430-fold compared with those in the infected mice treated with PBS (*P* < 0.001, n = 5). Notably, TNCs–Amo reduced the MRSA counts by 40-fold compared with the gold standard group (Van, *P* < 0.001, n = 5) [42]. Consequently, the TNCs–Amo treatment resulted in 90% recovery of the mice infected with MRSA compared with the control group (PBS) with 10% survival (Fig. 5d). Only 10% and 20% survival rates were observed for the groups treated with Amo and TNCs, respectively. These results revealed that the synergistic interaction between Amo and TNCs in this pneumonia model further emphasized the capacity of TNCs to eliminate the drug resistance of MRSA to β-lactam antibiotics and rescue mice from MRSA-induced pneumonia-related death.

To better understand the inflammation reaction in lung tissues, H&E staining was used after 2 and 8 days. Histopathological analyses showed that the lung alveoli of all groups were severely infected by MRSA, with massive inflammatory cells infiltrated, compared with the case of the healthy group 1 day post-nebulization (Fig. S19) [43, 44]. However, when we observed the thicknesses of the alveolar cavity walls, the lungs treated with Van and TNCs–Amo appeared to have thinner alveolar walls than the others. This result was consistent with the in vivo antibacterial results mentioned above. With the CFU of MRSA decreasing, the inflammation in the lungs disappeared gradually. Seven days after therapy, we observed that the state of the alveoli and the thickness of the alveolar walls in the TNCs–Amo group were the same as those in the healthy group, and few inflammatory cells were found in lung stroma compared with healthy lung stroma (Fig. 5e). Therefore, the MRSA-infected lungs recovered rapidly to their initial state after nebulization treatment with TNCs–Amo. For the PBS group, the alveolar space was replaced by abundant infiltration inflammatory cells with loss of alveolar structure. Although inflammation in the lungs of the monotherapy groups (Amo or TNCs) was not as serious as that in the PBS group, the monotherapy group had thicker alveolar walls and much more inflammatory cells than before. In addition, no signs of lesions were observed in the H&E staining of the brain, liver, heart, spleen, and kidney after 8 days for mice in the TNCs–Amo group (Fig. S20). Lung tissue histology supported the conclusion that TNCs and a β-lactam antibiotic can highly effectively eliminate MRSA infection in the lungs of surviving mice without side effects.

## 3 Conclusion

In this work, a natural product of TNCs zapped conventional β-lactam antibiotics back into action of treating MRSA pneumonia effectively in vivo through inhalation. We found a new mechanism to inhibit enzymes by a “pocket capping” effect. At present, small molecules inhibit enzymes mainly through organic compound molecules that bind to the active site on the enzyme to reduce protein activity or hinder biochemical reactions [45], [46], [47]. A single active site is easy to mutate, leading to failure of small molecules and the development of drug resistance [48]. The nanomaterial with right size is to completely cover the protein pocket through binding all residues around the protein pocket, and then entire active sites are locked in the protein pocket. Even if one or two active residues are mutated, it will not cause the nanomedicine to fail. Compared with small molecule inhibitors, nano-drug resistance develops a bit slower and nano-drugs have stronger activity to inhibit enzymes due to greater steric hindrance.

The great performance of biosafety research in piglets guaranteed the future clinical application of TNCs. TNCs are combined with β-lactam antibiotics to combat multi-drug resistant pathogen infections in addition to MRSA infections. The “pocket capping” effect may inspire further attempts to design new types of enzyme inhibitors in near future. These findings suggest that Chinese materioherbology may be a promising therapeutic strategy for pathogenic bacterial infected diseases.

## Declaration of Competing Interest

The authors declare that they have no conflicts of interest in this work.

## Appendix Supplementary materials

Image, application 1

Image, video 2
