# Supplementary material for: Using tea nanoclusters as β-lactamase inhibitors to cure multidrug-resistant bacterial pneumonia: A promising therapeutic strategy by Chinese materioherbology
Source: Fundam Res. 2021 Nov 25;2(3):496–504. doi: 10.1016/j.fmre.2021.11.019 (PMC11197604; doi:10.1016/j.fmre.2021.11.019)
Supplement: Supplementary file 1 [file mmc1.doc]

**Using tea nanoclusters as β-lactamase inhibitors to cure multidrug-resistant bacterial pneumonia: A promising therapeutic strategy by Chinese materioherbology**

Ziao Zhou^a,1^, Jun Li^b,1^, Lei Tan^a,1^, Xiangmei Liu^a,*^, Yufeng Zheng^c^, Zhenduo Cui^b^, Changyi Li^d^, Kelvin Wai Kwok Yeung^e^, Zhaoyang Li^b^, Yanqin Liang^b^, Shengli Zhu^b^, Shuilin Wu^b,*^

^a^*Biomedical Materials Engineering Research Center, Collaborative Innovation Center for Advanced Organic Chemical Materials Co-constructed by the Province and Ministry, Hubei Key Laboratory of Polymer Materials, Ministry-of-Education Key Laboratory for the Green Preparation and Application of Functional Materials, School of Materials Science & Engineering, Hubei University, Wuhan 430062, China*

^b^*School of Materials Science & Engineering, the Key Laboratory of Advanced Ceramics and Machining Technology by the Ministry of Education of China, Tianjin University, Tianjin 300072, China*

^c^*School of Materials Science & Engineering, State Key Laboratory for Turbulence and Complex System, Peking University, Beijing 100871, China*

^d^*Stomatological Hospital, Tianjin Medical University, No. 12, Qixiangtai Road, Heping District, Tianjin 300070, China.*

^e^*Department of Orthopaedics & Traumatology, Li Ka Shing Faculty of Medicine, The University of Hong Kong, Pokfulam, Hong Kong 999077, China*

*^1^ These authors contributed equally to this work.*

*Corresponding authors:

*E-mail* addresses: [shuilinwu@tju.edu.cn](mailto:shuilinwu@tju.edu.cn) (S.L. Wu)[; liuxiangmei1978@163.com](mailto:; liuxiangmei1978@163.com)(X.M. Liu)

**Table of Contents**

**Movie S1.** All atomic molecular dynamic simulation of TNCs-Amo-β-lactamase complex system

**Methods.**

**Figures**

**Fig. S1.** The reproducibility of the types and proportions of ingredients that make up TNCs.

**Fig. S2.**TIC mass spectra of TNCs

**Fig. S3.** Mass spectra of ingredients of TNCs

**Fig. S4.** Ion chromatograms of ingredients of TNCs

**Fig. S5.** Molecular formula of ingredients of TNCs.

**Fig. S6.** Zeta potential of the TNCs.

**Fig. S7.** Growth curves of bacteria, the Bliss Independence model

**Fig. S8.** CFU of MRSA treated with Amo at various concentrations combined with TNCs.

**Fig. S9.** Bacterial survival rate in antibacterial experiments

**Fig. S10.** FE-SEM images of bacteria.

**Fig. S11.** Confocal microscope images of MRSA treated with TNCs, Amo, and TNCs–Amo.

**Fig. S12.** The binding constant (K) of TNCs with β-lactamase was measured by ITC.

**Fig. S13.** TNCs-Amo-β-lactamase complex system

**Fig. S14.** Distance between samples and residues in β-lactamase active site

**Fig. S15.** The binding energy of TNCs and β-lactamase

**Fig. S16.** Cell viability of A549 cells treated by TNCs

**Fig. S17.** Hemolysis experiment images

**Fig. S18.** A549 cells coculture with MRSA

**Fig. S19.** H&E staining images of lungs in mice on day 2

**Fig. S20.** H&E staining images of liver, kidney, spleen, heart and brain in mice

**Methods**

**Materials.** Black tea was provided by Luyu International Group. Penicillin G potassium salt (Pen), Oxacillin sodium salt (Oxa), Ampicillin sodium salt (Amp), β-lactamase, Vancomycin (Van), Nitrocefin, Amoxicillin Sodium (Amo), Piperacillin Sodium (Pip) and Cloxacillin Sodium Salt Monohydrate (Clo) were purchased from Aladdin Reagent Co. Dialysis Membrane (1000) and Clavulanate Potassium (Cla) were purchased from Shanghai yuanye Bio-Technology Co. Ltd. NaCl and Ethanol were acquired from Sinopharm Chemical Reagent Co (China). Catechin (CA), Gallocatechin (GC), Catechin gallate (CG), Gallocatechin gallate (GCG), Epicatechin (EC), Epigallocatechin (EGC), Epicatechin gallate (ECG) and Epigallocatechin gallate (EGCG) were purchased from Solarbio life Sciences (China).

**Preparation of TNCs.** Black tea (6 g) was dispersed in deionized water (DI 60 mL). The mixture was put in a Teflon-lined stainless-steel autoclave (100 mL) at 85°C for 6 h. After the reaction, the suspension was centrifuged at 15000 r.p.m. for 30 min. And then the supernatant was filtered with 220 nm filter. After filtration, the solution was added into dialysis membrane (1000) and they were dialyzed for 3 days. The water was replaced by deionized (DI) water every 12 h. The process was over and the dialysate was centrifuged at 15000 r.p.m. for 1 h. And the solution was evaporated to less than 10 mL in a water bath at 80°C. The remains were dried in vacuum at 65°C and stored under Ar. Every time before using, TNCs were dissolved in DI and treated with an ultrasonic homogenizer (JY92-ⅡN, Ningbo Scientz Biotechnology Co. China) under the power of 120 W for 20 min.

**Characterization of TNCs.** The morphology of TNCs was observed by high resolution transmission electron microscope (HR-TEM, JEM-2100F). The zeta potential of TNCs (1 mg mL^-1^) was measured by particle size distribution analyzer (Malvern).

**HPLC-MS-MS analysis.** High-performance liquid chromatography (HPLC, UltiMate 3000 RS, thermo fisher technology (China) co. LTD) was used for quantitative analysis of TNCs. 300 μL TNCs were accurately taken and filtered through a 0.22 μm filter membrane. Chromatographic column was Thermo Hypersil GOLD 100 × 2.1 mm, 1.9 μm. Flow rate was 0.3 mL min^-1^. Mobile phase A was 0.1% formic acid aqueous solution. Mobile phase B was 0.1% formic acid and acetonitrile. Needle washing solution was methanol. Column oven temperature was 35°C. For mass spectrometer (Q Exactive high resolution mass spectrometer, thermo fisher technology (China) co. LTD) determination, ion source was electrospray ionization source (ESI). Scanning method was positive and negative ion switching scan. Detection method was full mass dd-MS_2_^-1^. Resolution was 70000 (full mass) and 17500 (dd-MS_2_). Scan range was 100.0 ~ 1500.0 m z^-1^. Electrospray voltage (Spary Voltage) was 3.8 kV (Positive). Capillary temperature was 300℃. The database (mzCloud, mzVault, Chem Spider) was retrieved and compared to the collected data.

**Bacterial strains and growth conditions.** Methicillin-resistant *Staphylococcus aureus* (MRSA CCTCC 16465) was provided from China Center for Type Culture Collection. The MRSA was cultured in the standard Luria-Bertani (LB) culture medium and at 37^o^C.

**Minimal inhibitory concentration (MIC) assay.** The standard method determining the MIC of the MRSA was on the basis of the method provided by the Clinical and Laboratory Standard Institute [1]. The MIC of TNCs, Pen, Oxa, Amp, Amo, Pip and Clo were measured to be 64, 16, 512, 8, 16, 128 and 2048 μg mL^-1^, respectively.

***In vitro* antibacterial kinetics assay.** As for Amo, the concentrations of Amo were 1×MIC and 2×MIC and the concentration of TNCs was 64 μg mL^-1^ all the time. The experiment groups were divided as TNCs (64 μg mL^-1^), Control (no treatment), Amo (1×MIC or 2×MIC) and TNCs-Amo (64 μg mL^-1^, 1×MIC or 2×MIC). Every 4 h subsequently until 12 h and 24 h, all groups were treated with MRSA (2×10^6^ CFU mL^-1^) and 10 μL diluted solution of each group was spread on solid LB agar plates and then the plates were put in an incubator at 37°C for 24 h. The number of colony-forming units (CFU) was considered as estimating the statement of MRSA growth. These antibiotics, Pen, Oxa, Amp, Amo, Pip and Clo were also treated with MRSA (2×10^6^ CFU mL^-1^) and the used concentration of all the antibiotics and TNCs was 1×MIC and 64 μg mL^-1^, respectively. After 8 h, the statistical method was the same as above.

**Compared to clinical inhibitors.** The experiment consisted of 6 groups and they were Control (no treatment), Amo, Cla, TNCs, Cla-Amo and TNCs-Amo. The concentrations of Amo, Cla, TNCs, Cla-Amo and TNCs-Amo were 32 μg mL^-1^, 64 μg mL^-1^, 64 μg mL^-1^, 64 μg mL^-1^-32 μg mL^-1^ and 64 μg mL^-1^-32 μg mL^-1^, respectively. All samples were treated MRSA (1×10^7^ CFU mL^-1^) suspension. After incubation for 8 h, 10 μL diluted solution of each sample was spread on solid LB agar plates and then the plates were put in an incubator at 37°C for 24 h. The CFUs of samples were counted to evaluate the statement of MRSA.

**Definition and calculation of synergism.** For Amo, Pen and Oxa, the used concentrations of the three antibiotics were 1, 0.5, 0.25, 0.125 and 0.0625×MIC. The antibiotics were combined with TNCs (64, 32, 16, 8 or 4 μg mL^-1^). All the groups were co-cultured with MRSA (5×10^5^ CFU mL^-1^) for 24 h. 10 μL diluted solution of each group was spread on solid LB agar plates and then the plates were put in an incubator at 37°C for 24 h. The CFU of groups were counted to evaluate the statement of MRSA.

According to the Bliss Independence model [2], *S* parameter of MRSA under antibiotic combinations was defined as:


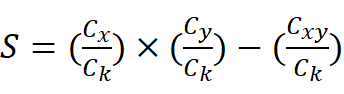


Where *C_x_* was only antibiotic group, *C_k_* was no treatment, *C_y_* was only TNCs group and finally *C_xy_* was the combination group of antibiotics and TNCs.

The survival rate of all groups was calculated from data of the spread plate. The survival rate was defined as Survival (%) = a/b×100%, b was the CFU number of the group without treatment and a was the CFU number of the treated groups.

**SYTOX Green bacterial membrane permeability assay.** Membrane permeability was investigated by SYTOX Green (Invitrogen) after treatments. Phosphate buffer saline (PBS) was used to wash MRSA three times. The experiment groups were divided as the Control, TNCs (64 μg mL^-1^), Amo (32 μg mL^-1^) and TNCs-Amo (64 μg mL^-1^-32 μg mL^-1^). These groups were treated with MRSA (5×10^7^ CFU mL^-1^) for 8 h. 100 μL solution of each group were combined with 100 μL solution of SYTOX Green (5 μM). They were incubated for 25 min at room temperature without light. Finally, the fluorescence intensity of these groups was measured by using a microplate reader (Spectra Max i3, Molecular Devices) with the excitation and emission wavelengths of 485 nm and 510~750 nm (2 nm interval), respectively. The images could be observed by Laser scanning confocal microscope (Nikon, AIR+).

**Characterization of bacterial morphology.** Transmission electron microscope (TEM, Hitachi HT770, Japan) and field emission scanning electron microscope (FE-SEM, Sigma 500, Germany) were employed to observe the bacterial morphology. MRSA bacteria were prepared as described in the section of “*In vitro antibacterial kinetics assay*”. For FE-SEM samples, the four groups were washed with phosphate buffered saline (PBS) three times and then fixed with 2.5% glutaraldehyde solution for 2 h. Next the groups were dehydrated in a series of alcohol gradient solutions (10 min each; 30, 50, 70, 80, 90 and 100%). And for TEM samples, the four samples were immersed in 2.5% glutaraldehyde solution for 2 h and then fixed with 1% osmic acid for 2 h at 4^o^C. These samples were washed with PBS three times and dehydrated in a series of alcohol gradient solutions (10 min each; 30%, 50%, 70%, 80% and 90%, 100% for 2×15 min). The samples were changed into ultrathin sections (60~70 nm) and immersed in 2% uranium acetate solutions for 15 min and lead citrate for 20 min.

**Enzyme activity testing assay.** For TNCs, the concentrations of 0, 16, 32, 64, 128 and 256 μg mL^-1^ were combined with β-lactamase of 1×10^4^ U mL^-1^ and then the mixtures were put into an incubator at 37°C for 8 h. After co-incubation, nitrocefin (250 μg mL^-1^) was added into the mixtures and co-cultured for 5 min. The optical density at 485 nm of the suspension was then determined with a spectrophotometer. Enzyme activity (%) was defined as:


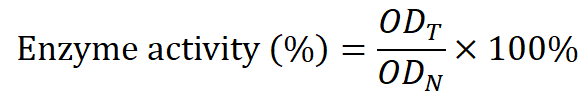


where OD_T_ was the optical density (OD) of tested samples and the OD_N_ was the OD value of the β-Lactamase group alone (no treatment).

**Isothermal Titration Calorimetry.** The interaction between the TNCs and β-lactamase was measured by MicroCal VP-ITC Isothermal Titration Microcalorimeter (Malvern Panalytical). Experimental temperature was 25°C (298K). Syringe (290 µL)-sample E (β-lactamase) concentration was 0.3 mM, Sample pool (1.4 mL)-sample TNCs concentration was 0.02 mM (0.1 mM original solution diluted); rotation speed was394 rpm; Titration procedure: 28 drops, the first drop was 2 µL and the second to 28 drops was 10 µL, the interval between each drop was 180 s.

**Model construction and calculation method.** The β-lactamase protein (3BLM) was from the Protein Databank (PDB, https://www.rcsb.org/). For the crystal structure of β-lactamase, we used ADT to assign AD atom type to it, then hydrogenate, add charge, and determine the protonation state of the active site residue for subsequent molecular docking simulation calculations. According to the results of HPLC-MS-MS and the structure of each small molecule, the HEX8.0 program was used to dock CA, GC, CG, GCG, EC, EGC, ECG and EGCG, respectively. In the docking process, select the Shape+Electro+DARS mode when searching for the conformation of the small molecule-small molecule complex, and other input parameters are in the default state. and then the HEX8.0 program was used to calculate the lowest energy mode among these small molecules [3]. Finally, the possible interaction modes and structural models of these small molecules forming tea polyphenols nanocluster complexes (TNCs) were predicted.

The active sites of β-lactamase have been reported and bound to β-lactam antibiotics through key residues SER70, LYS73, GLU166 and ASN170 [4, 5]. Therefore, these active sites were selected as the binding sites of amoxicillin sodium (Amo) and TNCs with β-lactamase. β-lactamase was set as the receptor. TNCs and Amo were set as ligands. Auto-Dock was used for molecular docking calculation.

**Molecular dynamics simulation of TNCs, Amo and** **β-lactamase.** First, TNC, Amo, and β-lactamase complex system was constructed by Packmol [6]. TNC, Amo and β-lactamase had a positive triangle structure, making TNCs, Amo and the enzyme be the same distance and then a complex structure of TNCs, Amo and β-lactamase was formed. Then use Gromacs2018 to perform molecular dynamics simulation of the complex system to study the dynamic behavior of the combination of the two with enzymes. In the dynamic simulation process, the GROMOS 53A6 force field is used for the nanoparticles, and the SPC model is used for the water molecules. After the system is built, use the steepest descent method and the conjugate gradient method to optimize the energy of the system to reach the best state. In order to adapt the system to the simulated environment, constant temperature (NVT) ensemble balance and constant pressure (NPT) ensemble balance are performed [7]. In the balance process, the temperature coupling adopts the V-rescale method, and the thermal coupling time constant is 0.1 ps. Then the nanoparticle system is simulated for 15 ns, the simulation uses the leapfrog algorithm, the integration step is set to 2fs, the long-range electrostatic interaction is processed by the PME algorithm, the short-range Coulomb cut-off radius is set to 1.2 nm, and the van der Waals effect is calculated The truncation radius is 1.2 nm. The system adopts periodic boundary conditions in all directions, and uses the LINCS algorithm to constrain the bond length of protein and lipid molecules.

***In vitro* cytotoxicity assay.** Cytotoxicity was tested with lactate dehydrogenase (LDH) cytotoxicity assay kit (Yeasen Biotech Co. China). Human alveolar basal epithelial A549 cells (ATCC) were used to determine the cytotoxicity of samples. The F12K medium used to culture A549 cells were combined with 10% (v/v) fetal bovine serum (FBS) and 1% penicillin-amphotericin solution (HyClone). The cells were cultured at 37°C in atmosphere with 5% CO_2_. There were two experiments to conduct. One for pure TNCs, A549 cells (10^4^ cells cm^-2^) were cultured in 96-well plate for 24 h and then combined with a series of concentrations of TNCs (128, 256, 512, 1024 and 2048 μg mL^-1^). They were co-cultured in 96-well plate for 1 day, 3 days and 7 days. In these days, LDH were added to the samples to measure the cytotoxicity. Another for TNCs-Amo, the experiment was divided into the Control (no treatment), TNCs (128 μg mL^-1^), Amo (128 μg mL^-1^) and TNCs-Amo (128 μg mL^-1^-256 μg mL^-1^). A549 cells were incubated in 96-well plate for 24 h before adding samples. The four groups were added into the cell well. After incubation for 1 day, 3 days and 7 days, the well of each group was measured using LDH assay kit.

**Hemolytic measurement.** The blood from New Zealand rabbits was used to measure the hemolytic ability of samples. 2 mL blood was added into 20 mL saline (0.9% NaCl) and then shaken evenly. The solution was centrifuged (1500 r.p.m., 10 min) to remove the supernatant. The precipitated red blood cells were washed three times with physiological saline as the described above until the supernatant did not appear red. The obtained red blood cells were mixed with physiological saline solution to prepare a 2% suspension for testing. The experiment was divided into PBS (negative control), 1% TritonX-100 (positive control), TNCs (256 μg mL^-1^), Amo (512 μg mL^-1^) and TNCs-Amo (256 μg mL^-1^-512 μg mL^-1^). The five groups were combined with red blood cells solution and cultured at 37°C. After the incubation for 3 h, the supernatant was aspirated and added to a 96-well plate and the absorbance was measured at a wavelength of 545 nm. Hemolytic rate was calculated according to the standard [8].

**MRSA and A549 cells co-culture.** A549 (10^4^ cells cm^-2^) cells were seeded as the above. MRSA (5×10^6^ CFU mL^-1^) were added to A549 cells in the same medium without 1% penicillin-amphotericin solution. The wells were combined with four groups. They were the Control (no treatment), TNCs (64 μg mL^-1^), Amo (16 μg mL^-1^) and TNCs-Amo (64 μg mL^-1^-16 μg mL^-1^). After incubation for 24 h, the cytotoxicity of A549 cells was determined by LDH assay kit. And 10 μL diluted suspension of each sample was spread onto the surface of solid LB agar plates and the plates were cultured at 37°C for 24 h. Cell viability (%) was defined as:


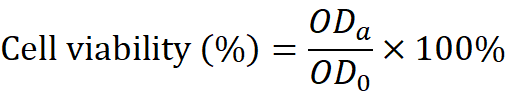


where OD_a_ was the optical density (OD) of tested samples and the OD_0_ was the OD value of the control group (no treatment).

**Piglet models of biosafety.** All animal experiments were conducted under an approved guideline of the Institutional Animal Care and Use Committee. The experiments were conducted in the Tianjin hospital of ITCWM Nankai hospital, China. The female Bama piglets were divided into two groups (n=3) at random. One was healthy without treatment and the other was nebulized with TNCs-Amo (64 μg mL^-1^-40 μg mL^-1^). The time of nebulization was 15 min and twice a day. After 7 days of treatment, the piglets were sacrificed and the blood samples of piglets were examined for hematology (MCV, MPV, HCT, RDW, WBC, PLT, RBC, MCHC and HGB), liver function (TP, ALB, ALP, ALT, CHE, AST, GGT, DBIL), kidney function (BUN, CRE, UA). We used hematoxylin-eosin (H&E) staining to investigate the toxicology of brain, heart, spleen, lung, kidney and liver.

**MRSA-induced pneumonia model.** All animal experiments were conducted under an approved guideline of the Institutional Animal Care and Use Committee. The experiments were conducted in the Tianjin hospital of ITCWM Nankai hospital, China. Seven-to-eight-week old male BALB/c mice were used in MRSA-induced pneumonia model. Firstly, MRSA were seeded in the liquid medium for 24 h and then the MRSA were centrifuged (7000 r.p.m., 10 min) and the MRSA were washed with PBS three times. According to the method of building a lung model of infection [9], a lethal dose (5×10^8^ CFU) of MRSA that 60% of mice (*n*=10) were killed by MRSA was introduced into mice. The experiment was divided into the Control (PBS), Van (25 mg mL^-1^), Amo (40 μg mL^-1^), TNCs (64 μg mL^-1^) and TNCs-Amo (64 μg mL^-1^-40 μg mL^-1^). The nebulization time was 15 min for all groups each time. After 1 day of infection, the nebulizer was loaded with the four samples, the Control (PBS), Amo (40 μg mL^-1^), TNCs (64 μg mL^-1^) and TNCs-Amo (64 μg mL^-1^-40 μg mL^-1^) and nebulized mice with the four samples twice. The mice were weighed and injected intravenously at a dose of 250 mg kg^-1^. The Van concentration was 25 mg mL^-1^, and if the weight of mouse was 25 g, 6.25 mg Van was injected, that is, 0.25 mL of 25 mg mL^-1^ Van was injected. The Van injection was performed by tail vein after 1, 3, 7 days of infection, respectively. After 2 days of infection, the number of the CFU of alveolar lavage fluid was determined *via* using spread plate method. H&E staining was performed to analyze histopathology of lung. On the day 2 and 8, the lung conditions of samples were investigated by H&E staining. Meanwhile, we used H&E staining to investigate the toxicology of brain, heart, spleen, kidney and liver.

**Statistical analysis.** The obtained data were determined based on Origin 8.5 and GraphPad Prism software, using a one-way classification of ANOVA and two-tailed heteroscedastic Student’s *t*-test, where differences were considered as statistically significant with possibility *P* < 0.05. All experiments were conducted according to biological repetition.


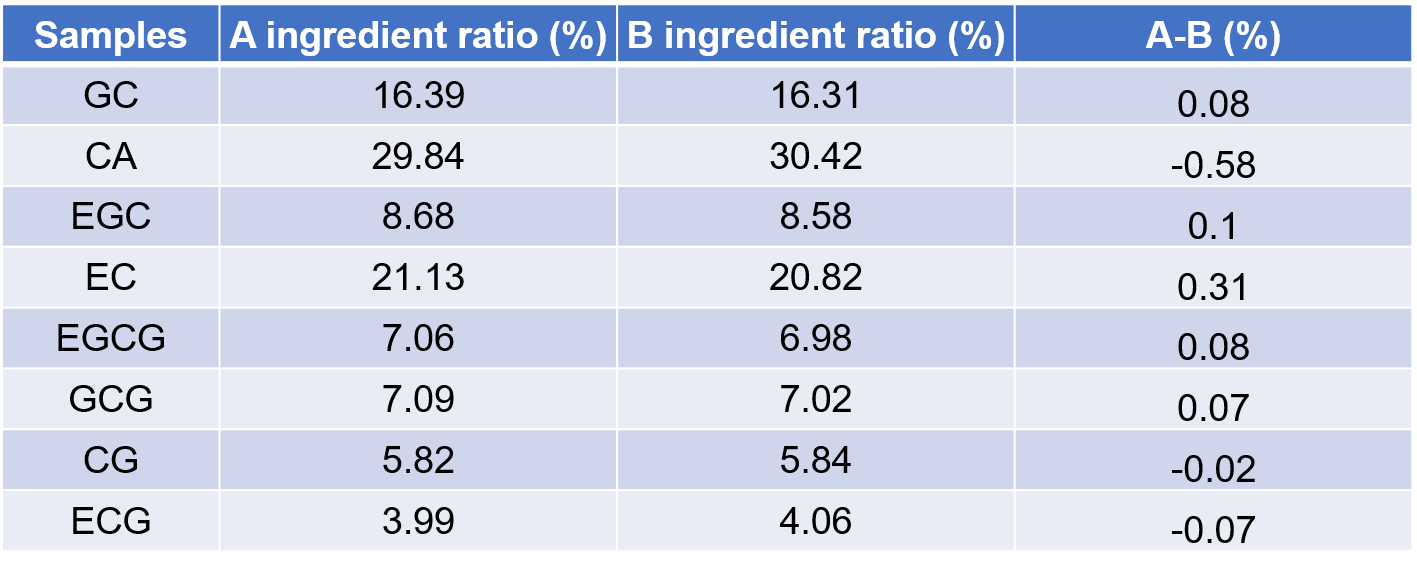


**Fig. S1.** The reproducibility of the types and proportions of ingredients that make up TNCs.


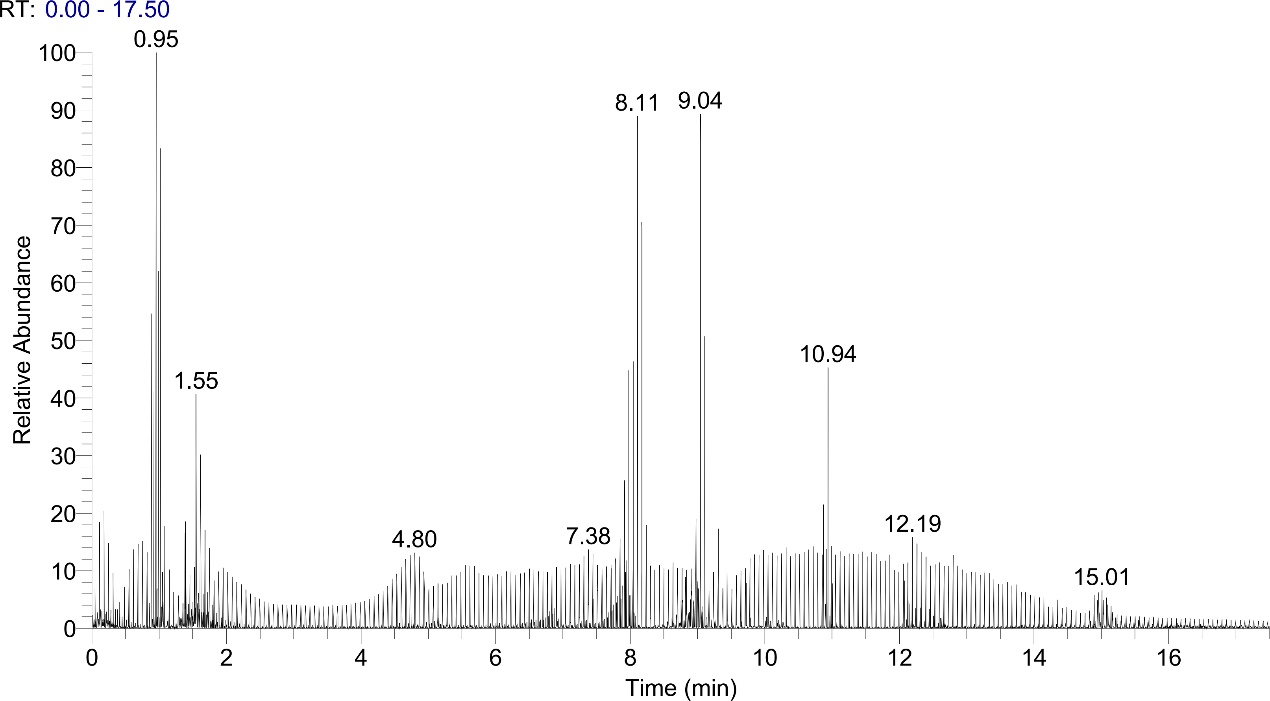


**Fig. S2.** TIC mass spectra of TNCs.

.


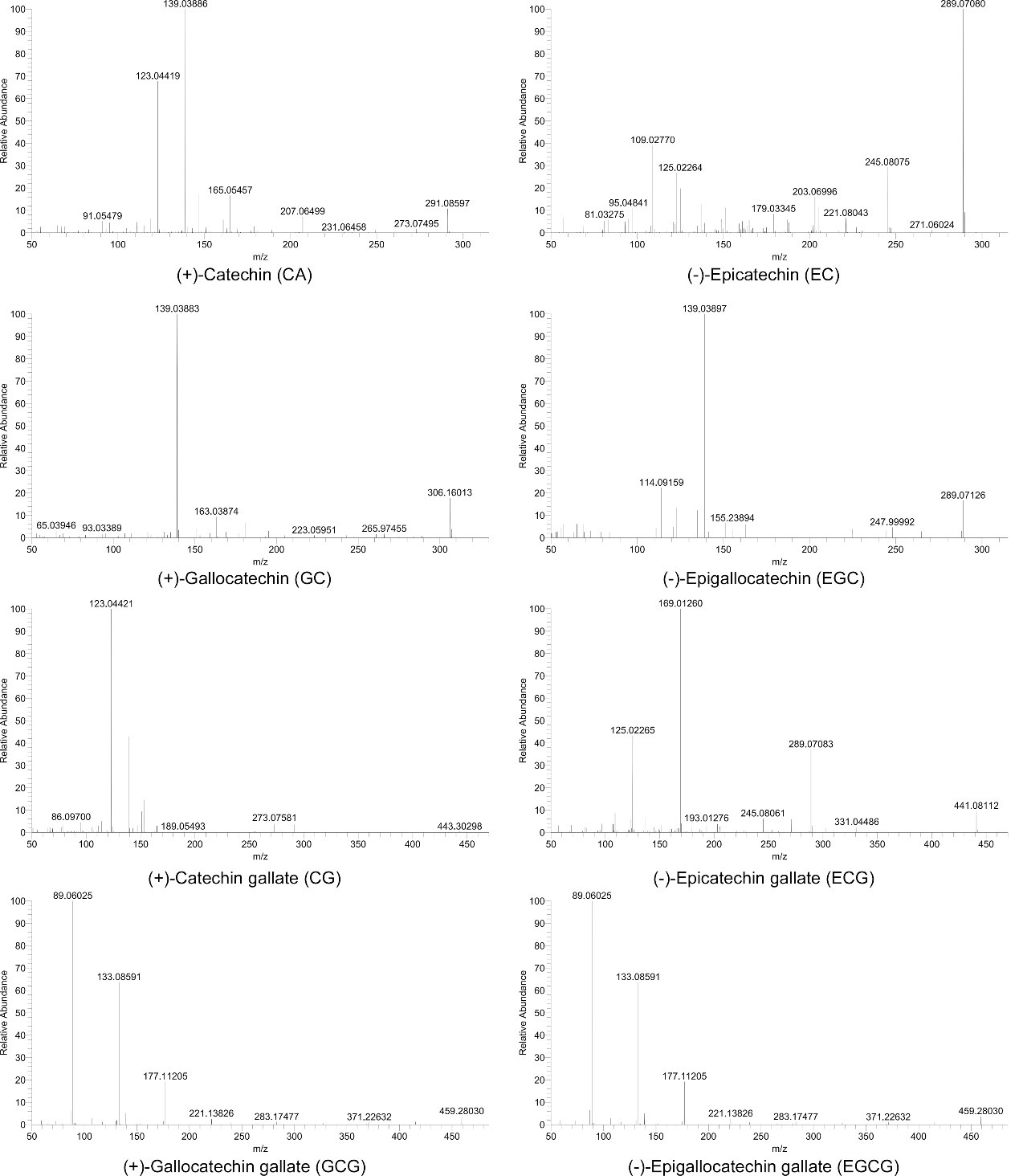


**Fig. S3.** Mass spectra of the main ingredients of TNCs (CA, GC, CG, GCG, EC, EGC, ECG and EGCG)


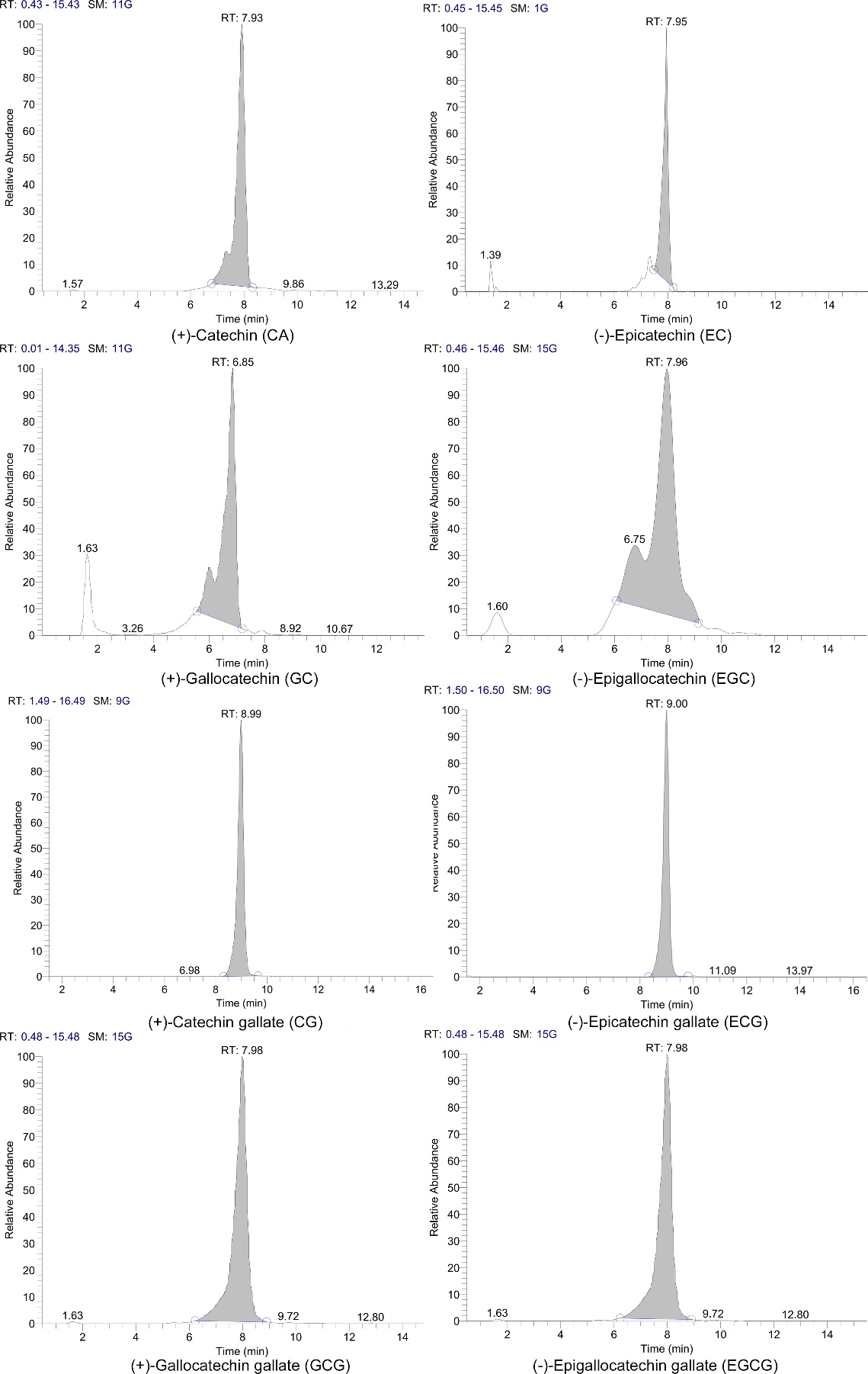


**Fig. S4.** Ion chromatograms of the main ingredients of TNCs (CA, GC, CG, GCG, EC, EGC, ECG and EGCG)


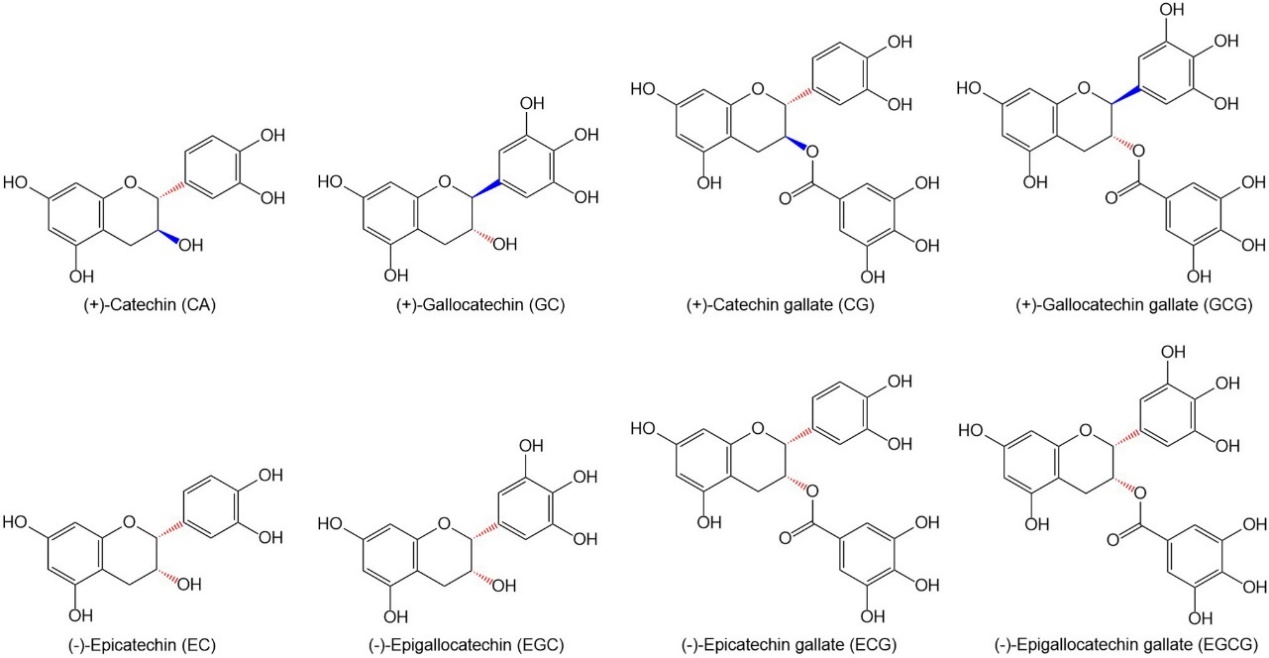


**Fig. S5.** Molecular formula of the main ingredients of TNCs (CA, GC, CG, GCG, EC, EGC, ECG and EGCG)


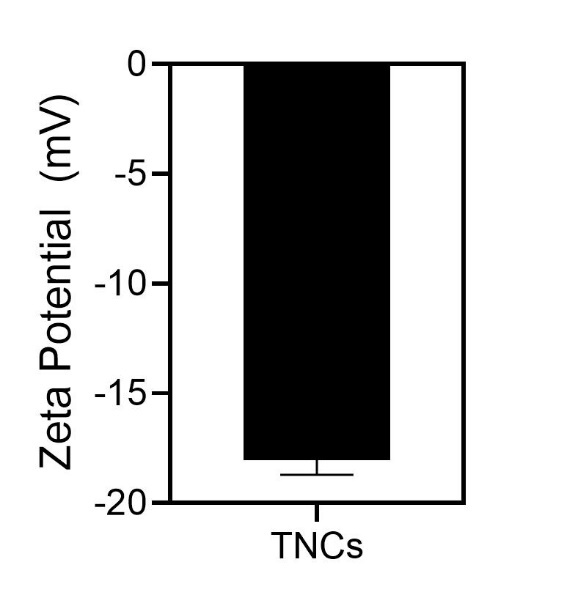


**Fig. S6.** Zeta potential of the TNCs. Zeta potential of the TNCs was about -18 mV.


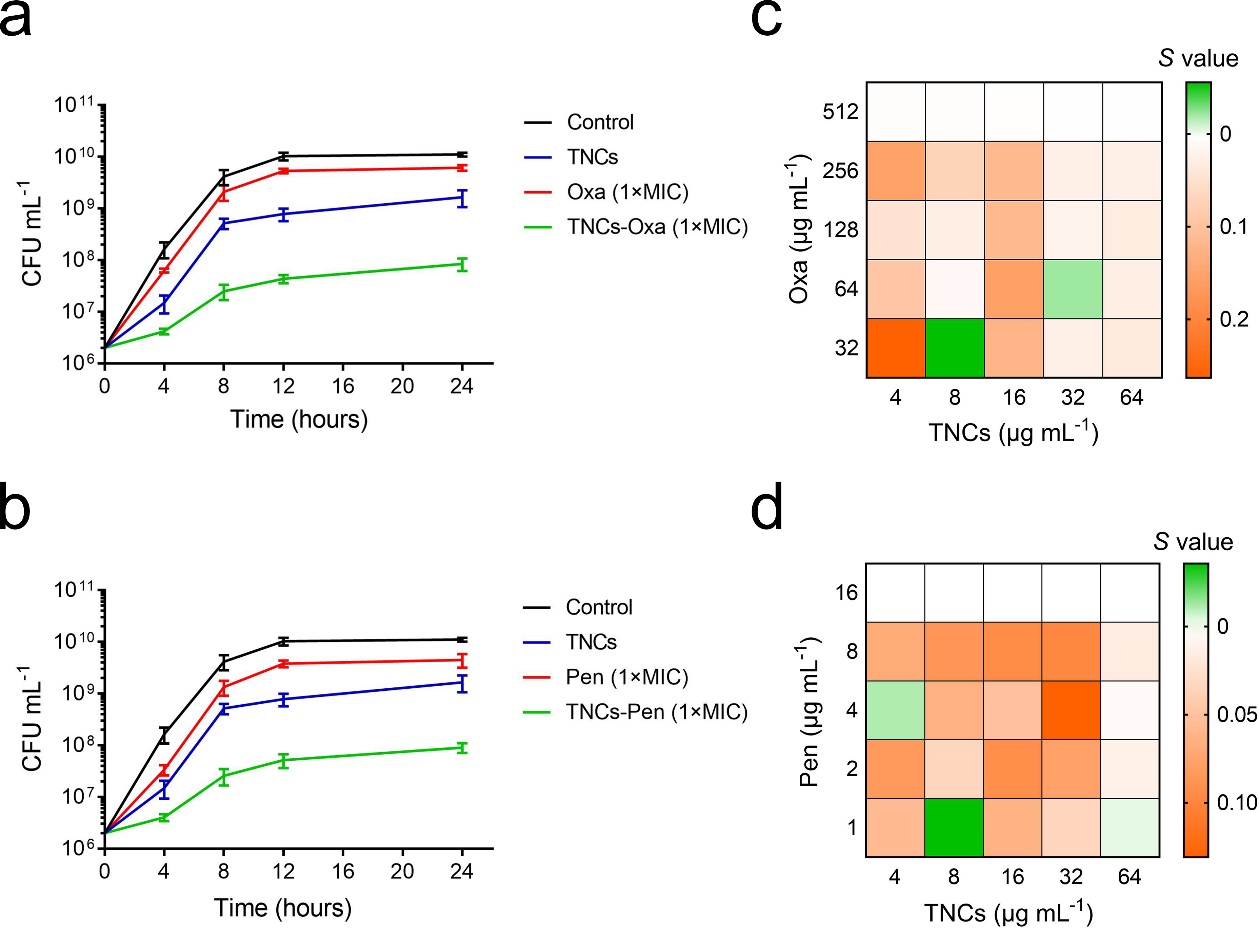


**Fig. S7. a,** Growth curves of bacteria under the treatment with TNCs, Oxa (1×MIC) and TNCs-Oxa (1×MIC) for 24 h. **b,** Growth curves of bacteria under the treatment with TNCs, Pen (1×MIC) and TNCs-Pen (1×MIC) for 24 h. **c, d,** The Bliss Independence model is used to evaluate the synergistic interaction between TNCs and antibiotics (Oxa, Pen). *S* > *0* indicates the synergistic interaction and *S* < *0* represents the antagonistic interaction. n = 3 biologically independent experiments.


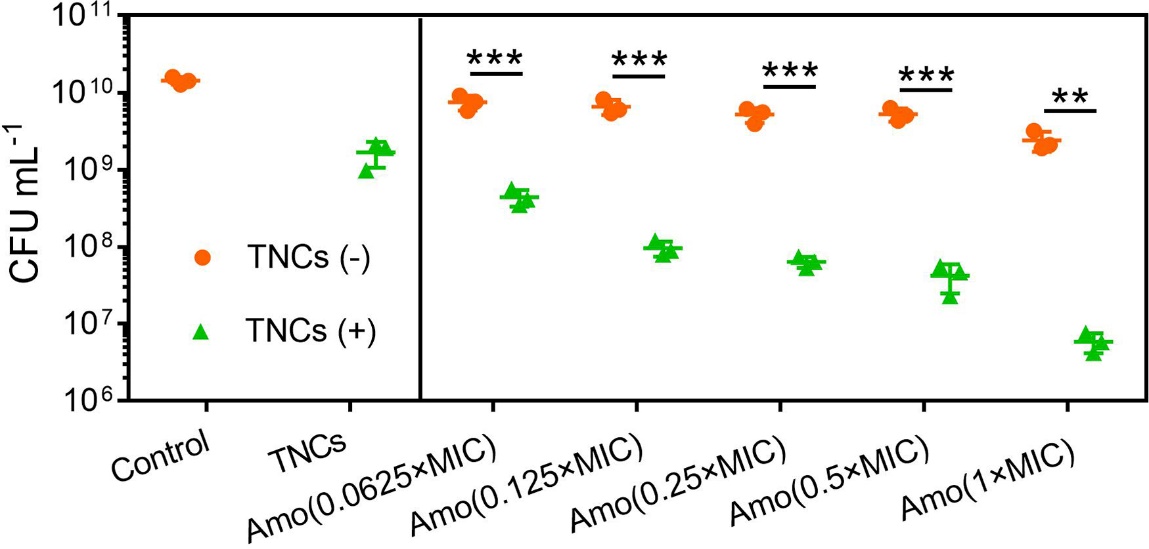


**Fig. S8.** CFU of MRSA treated with Amo at various concentrations combined with TNCs, cocultured for 24 h. n = 3 biologically independent experiments. ***P* < 0.01, ****P* < 0.001.


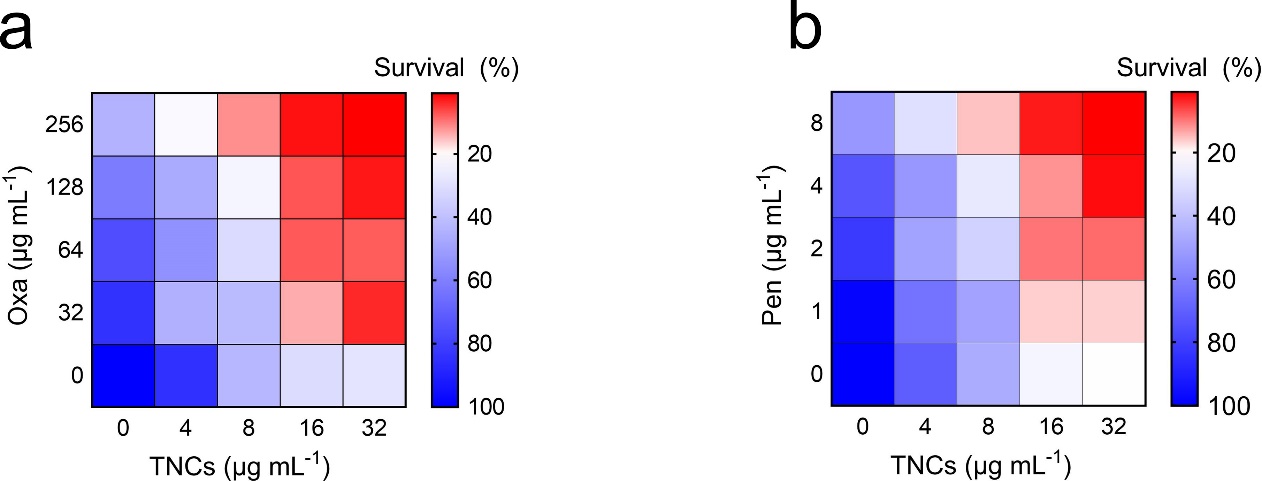


**Fig. S9. a, b,** Bacterial **s**urvival rate treated with antibiotics (Oxa, Pen) at various concentrations combined with TNCs at various concentrations.


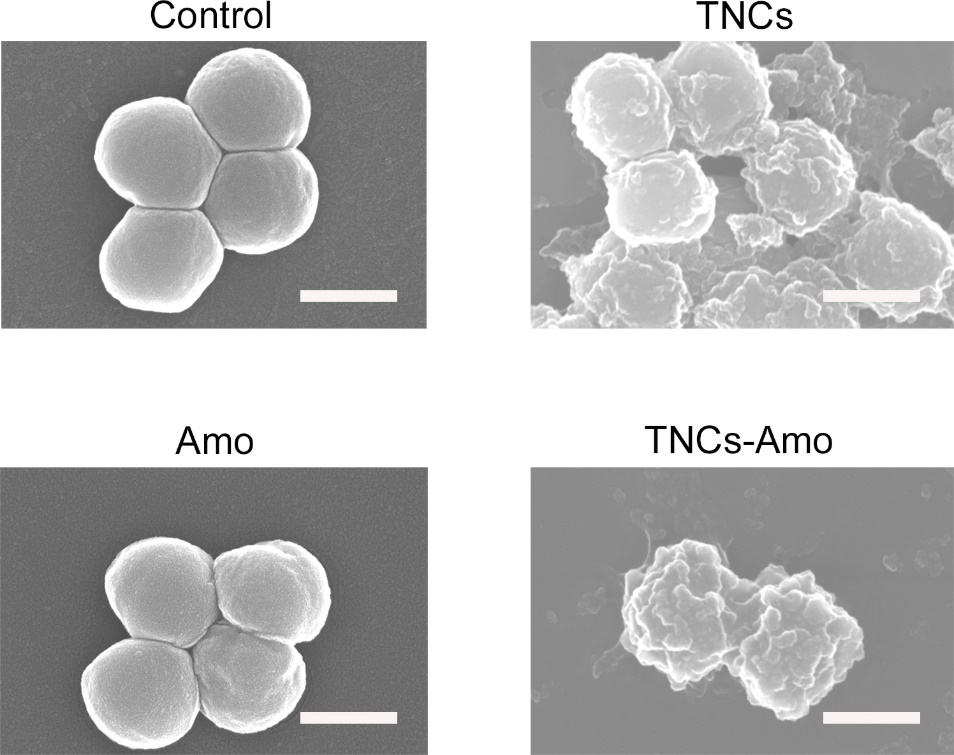


**Fig. S10.** Comparison of bacterial FE-SEM images in the presence of TNCs, Amo and TNCs-Amo. Scale bar, 500 nm.


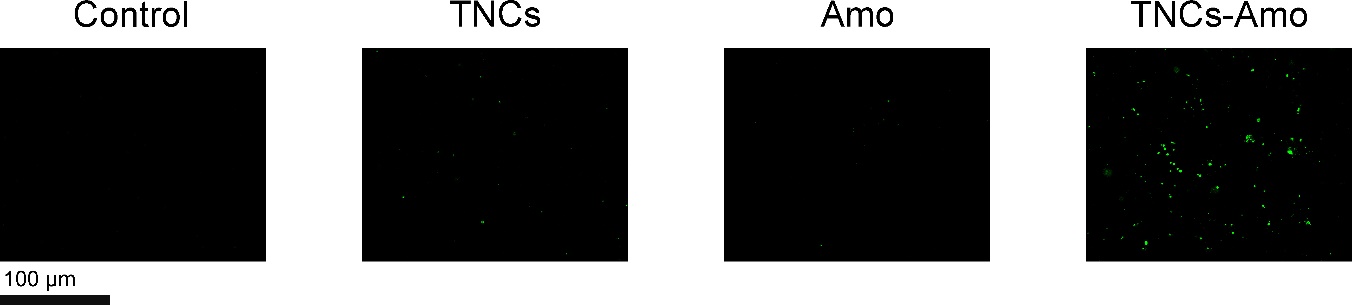


**Fig. S11.** Confocal laser scanning microscope images. MRSA treated with TNCs, Amo, and TNCs–Amo were stained by SYTOX Green.


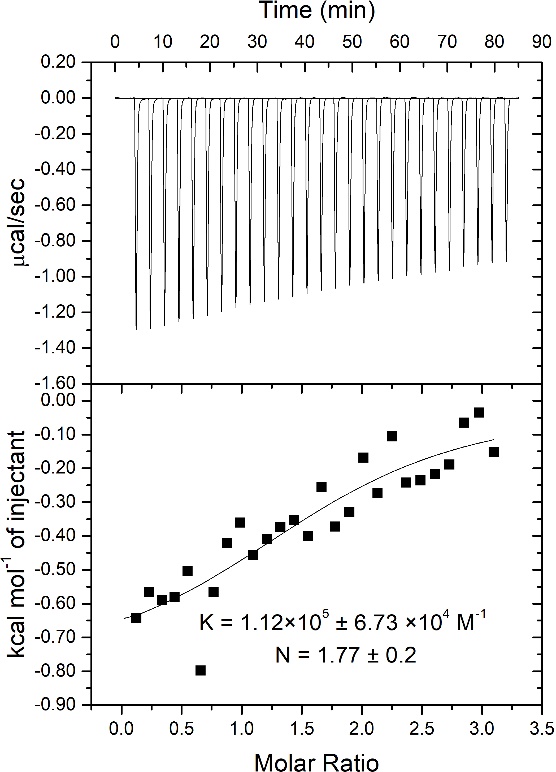


**Fig. S12.** The binding constant (K) of TNCs with β-lactamase was measured by ITC.


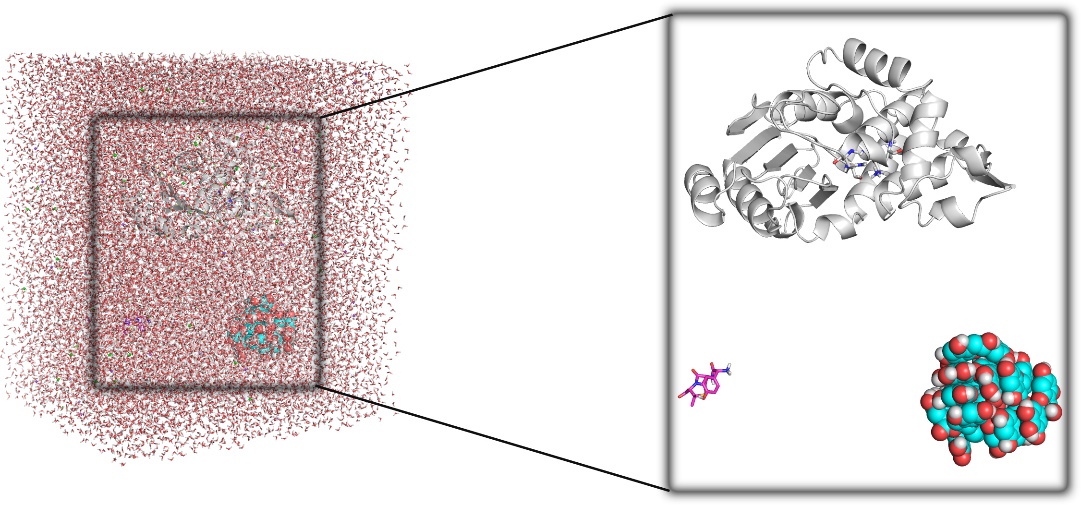


**Fig. S13.** TNCs-Amo-β-lactamase complex system


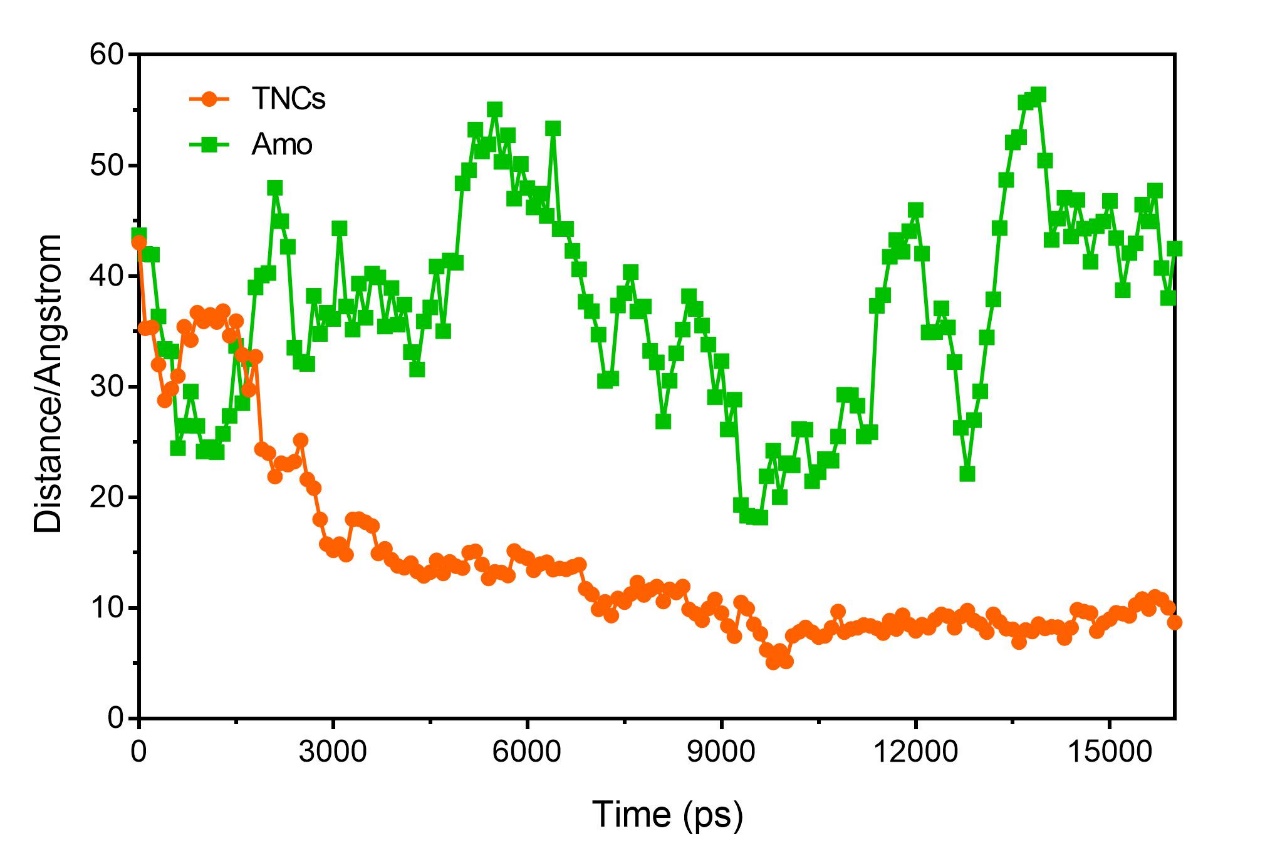


**Fig. S14.** Distance between samples (TNCs, Amo) and key residues in β-lactamase active site during molecular dynamics simulation.


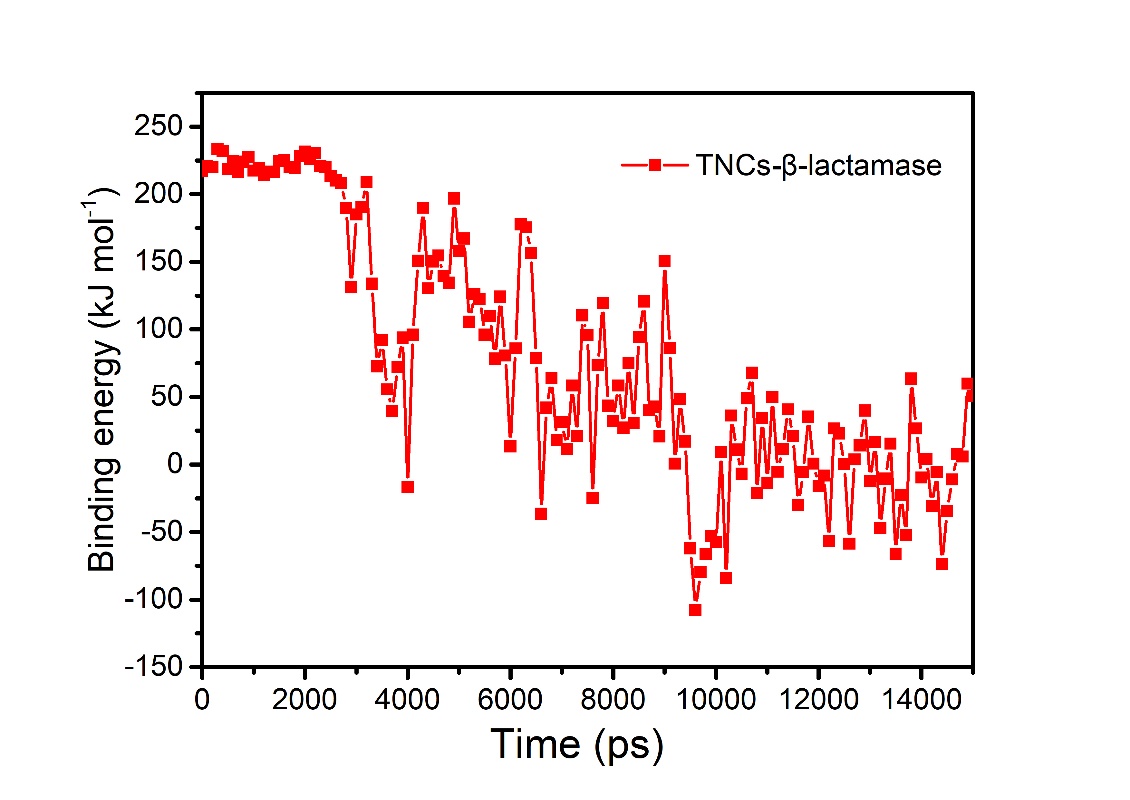


**Fig. S15.** The binding energy of TNCs and β-lactamase during molecular dynamics simulation.


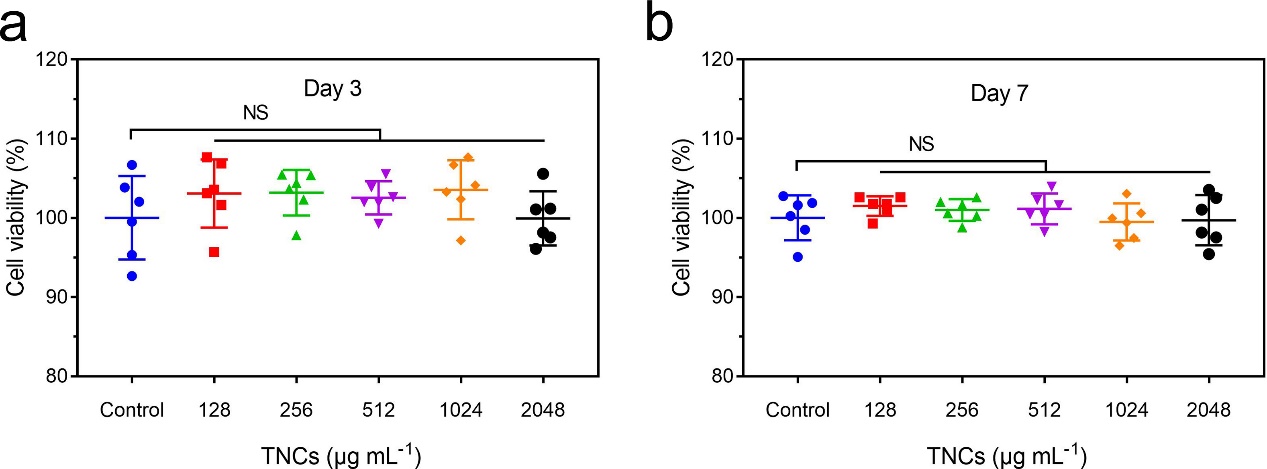


**Fig. S16. a, b,** Viability of A549 cells treated by TNCs at various concentrations after 3 and 7 days.


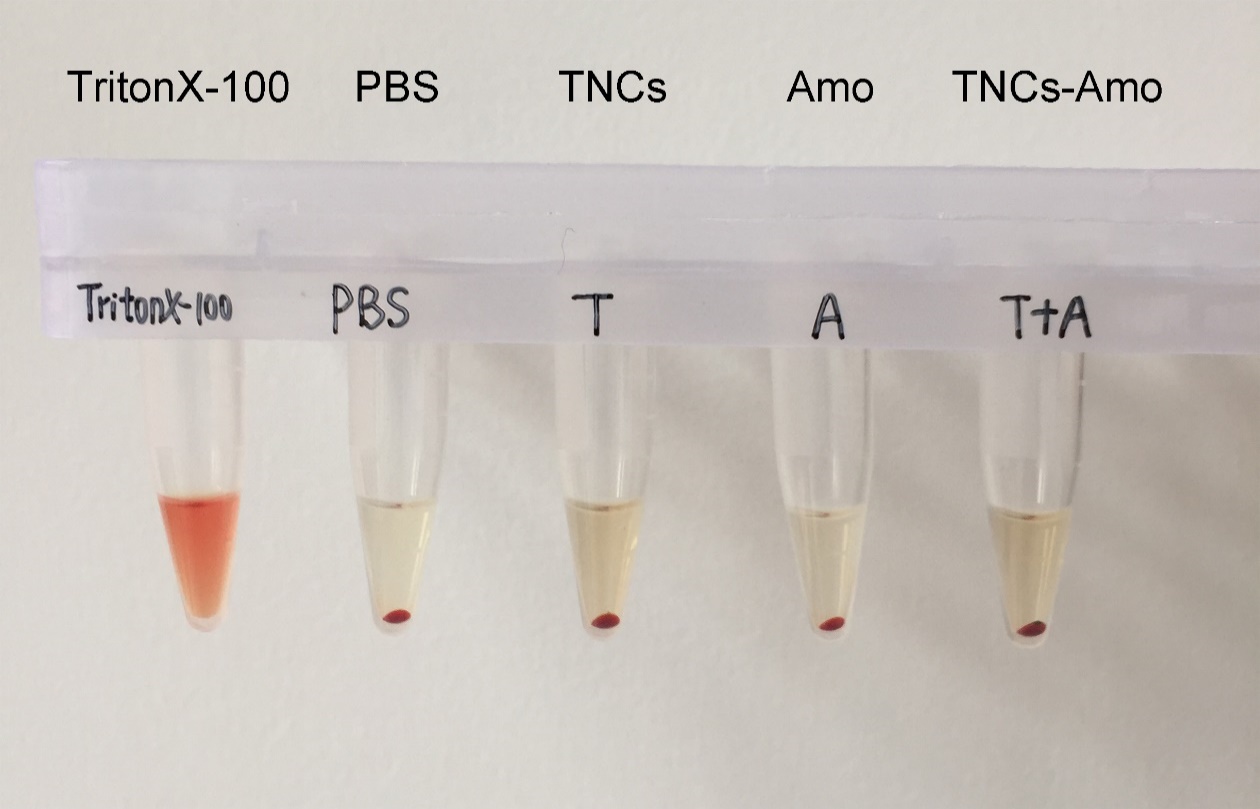


**Fig. S17.** Hemolysis experiment images of different groups.


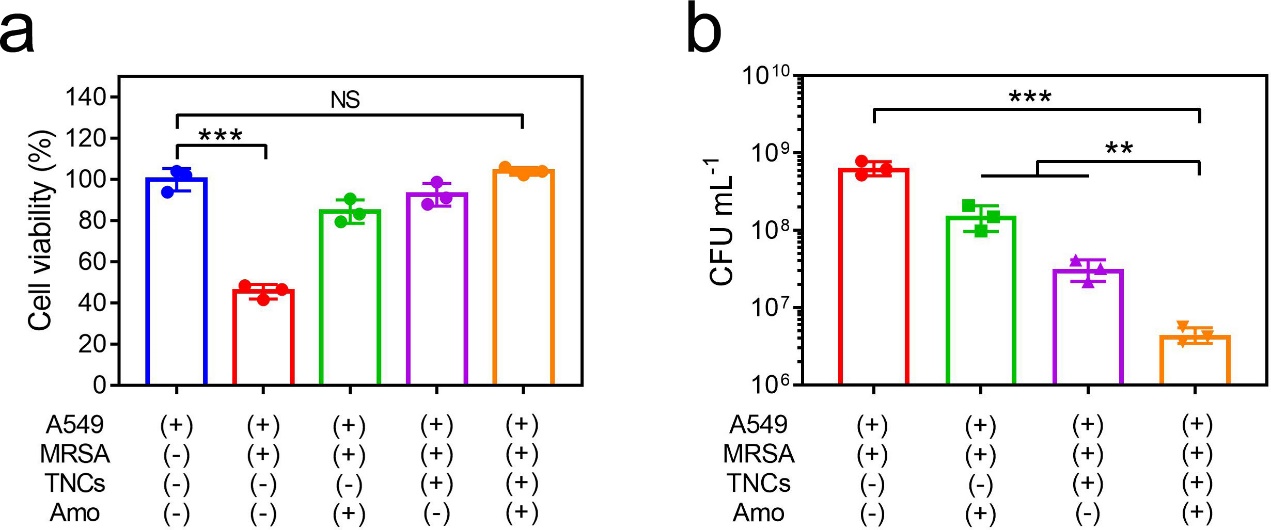
**Fig. S18. a,** Viability of A549 cells treated by samples (TNCs, Amo) and MRSA. **b,** CFU mL ^-1^ of MRSA treated with TNCs-Amo in the A549+MRSA coculture model after 24 h. n = 3 biologically independent experiments, ***P* < 0.01, ****P* < 0.001. The cell viability of the MRSA group decreased to below 50% compared with the A549 cells group alone. With the addition of both TNCs and Amo, the observed outcome was that the cell viability dramatically increased, and almost no difference was observed between the A549 cells group and the TNCs–Amo group. However, for MRSA, the CFU mL^-1^ of the combinatorial group was reduced by two orders of magnitude compared with that of the group without treatment.


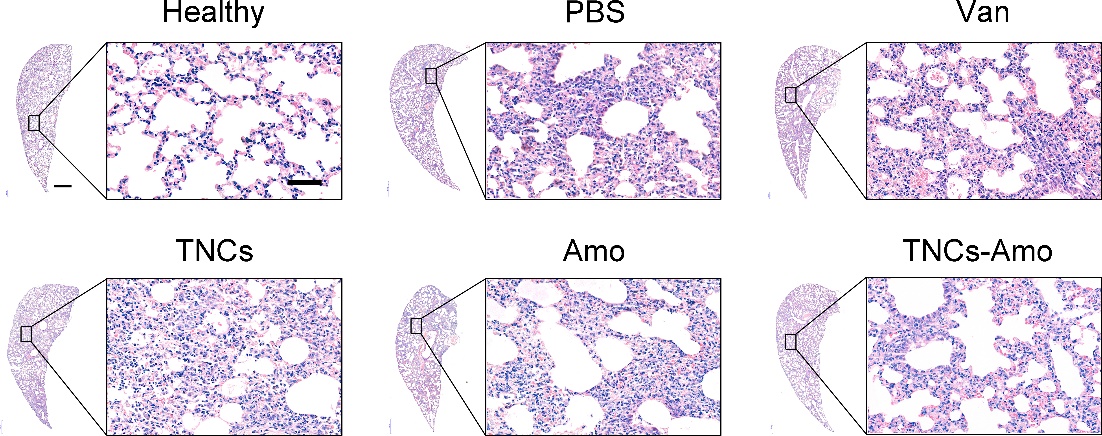


**Fig. S19.** H&E staining images of lungs in mice on day 2. Scale bar (left), 500 μm. Scale bar (right), 50 μm.


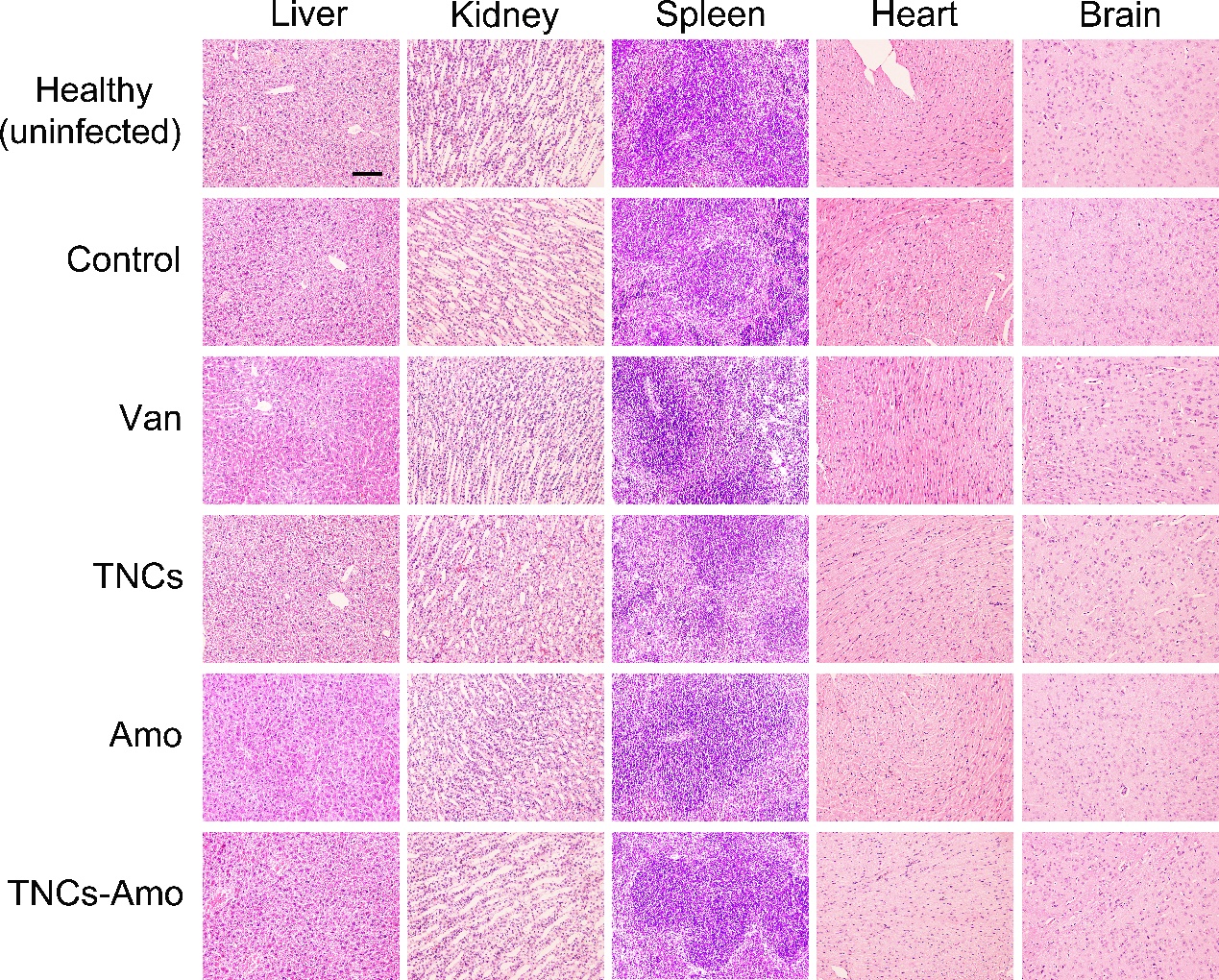


**Fig. S20.** H&E staining images of liver, kidney, spleen, heart and brain in mice on day 8. Scale bar, 100 μm.

**References**

1. Anon. CLSI. Methods for Dilution Antimicrobial Susceptibility Tests for Bacteria That Grow Aerobically; Approved Standard—Ninth Edition. CLSI document M07‐A9. (2012).
2. Courtney, C. M. et al. Potentiating antibiotics in drug-resistant clinical isolates via stimuli-activated superoxide generation. *Sci. Adv.* 3, e1701776 (2017).
3. Raharjo, S. J., Mahdi, C., Nurdiana, N., Kikuchi, T. & Fatchiyah, F. J. Binding Energy Calculation of Patchouli Alcohol Isomer Cyclooxygenase Complexes Suggested as COX-1/COX-2 Selective Inhibitor. Adv. Bioinform. f1-12 (2014).
4. Herzberg, O. & Moult, J. J. Bacterial resistance to beta-lactam antibiotics: crystal structure of beta-lactamase from Staphylococcus aureus PC1 at 2.5 A resolution. *Science* 236, 694-701.
5. Drawz, S. M. & Bonomo, R. A. Three decades of β-lactamase inhibitors. *Clin. Microbiol. Rev.* 23, 160-201 (2010).
6. Martínez, L., Andrade, R. A., Birgin, E. G. & Martínez, J. M. J. Packmol: A package for building initial configurations for molecular dynamics simulations. *J. Comput. Chem.* 30, 2157-2164 (2009).
7. Aragones, J. L., Valeriani, C. & Vega, C. J. Note: Free energy calculations for atomic solids through the Einstein crystal/molecule methodology using GROMACS and LAMMPS. *J. Chem. Phys.* 137, 146101.
8. Shu, J. L., O'Brien-Simpson, N. M., Pantarat, N., Sulistio, A. & Qiao, G. G. J. Combating multidrug-resistant Gram-negative bacteria with structurally nanoengineered antimicrobial peptide polymers. *Nat. Microbiol.* 1, 16162 (2016).
9. Hussain, S. et al. Antibiotic-loaded nanoparticles targeted to the site of infection enhance antibacterial efficacy. *Nat. Biomed. Eng.* 2, 95-103 (2018).
